# Structural and Gas Uptake Studies on Iron(II) β‑Diketiminate Metallomacrocycles

**DOI:** 10.1021/acs.organomet.5c00208

**Published:** 2025-08-01

**Authors:** Adam N. Barrett, Leah Webster, Danila Gasperini, Rémi Castaing, Mary F. Mahon, Ruth L. Webster

**Affiliations:** a Department of Chemistry, 1555 University of Bath, Claverton Down, Bath BA2 7AY, United Kingdom; b Yusuf Hamied Department of Chemistry, 2152 University of Cambridge, Lensfield Road, Cambridge CB2 1EW, United Kingdom; c Chemical Characterisation Facility, Research Infrastructure and Facilities, 1555 University of Bath, Claverton Down, Bath BA2 7AY, United Kingdom

## Abstract

Three iron metallomacrocycles were synthesized and characterized using single crystal X-ray crystallography. Varying the amide coligand allows for macrocycles containing three, four, or six iron centers. The metallomacrocycles show extended structures in the solid state that are reminiscent of metal-organic framework structures; thus, the adsorbative properties of the complexes in the solid state have been investigated using N_2_. Based on the data, three metallomacrocyles display classical type II isotherms and pore size distributions synonymous with mesoporous materials.

## Graphical abstract

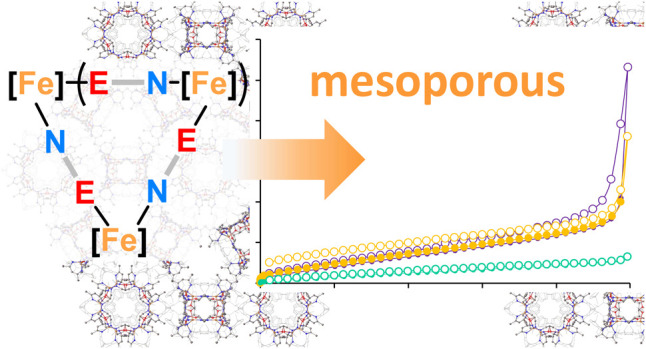

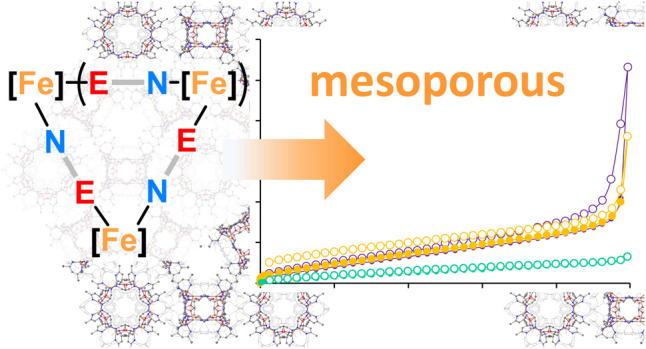

## Introduction

In the early 1990s, the first evidence of a metal-containing macrocycle was reported by Ogura and co-workers, consisting of palladium ethylenediamine (en) complexes bridged by 4,4′-bipyridine (Scheme 1a).^1^ This was followed by further tetrameric species comprising palladium and platinum 1,3-bis­(diphenylphosphino)­propane (dppp) complexes with the same bridging ligand.^2^ Since then, a plethora of different metallomacrocyclic species (or metallocrowns) has been presented with various transition metal and main group elements.^3,4^ Such examples have found application in molecular recognition, for example, the encapsulation of a range of aromatic species,^1,5,6^ and chemosensing of species such as small ions,^7^ nitroaromatics,^8^ and multicarboxylate anions.^9^ Further success for the use of metallomacrocycles has been found in cavity-controlled catalysis; select examples include Diels–Alder^10^ and acyl transfer-type transformations,^11,12^ as well as enantioselective ring-opening^13^ and cyclopropanation^14^ reactions, all of which have been facilitated within metallomacrocyclic ensembles. Applications for these species have also extended to pharmaceutical uses; for example, Therrien and co-workers have reported multiple investigations into anticancer activity of tetrametallic ruthenium and osmium metallomacrocycles.^15–17^

**Scheme 1:**
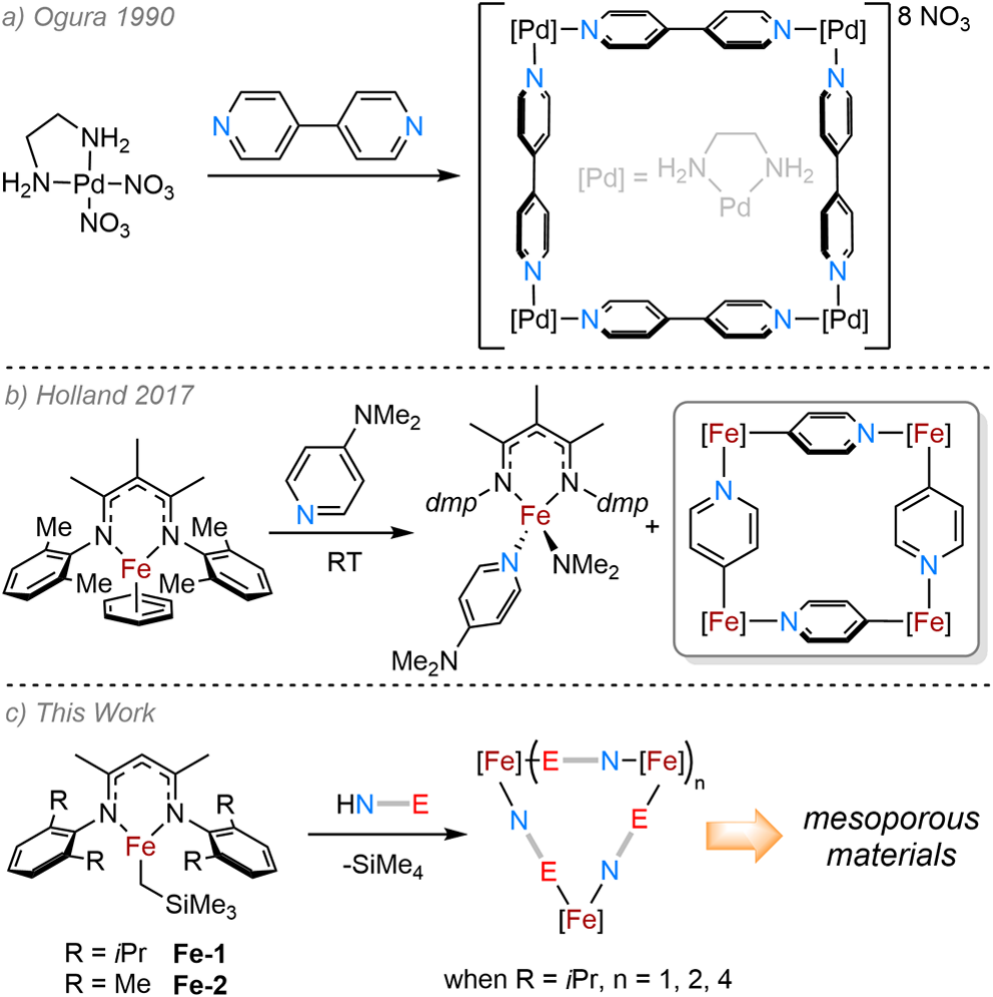
(a) Ogura and Co-Worker’s Synthesis of the First Metallomacrocyclic Species; (b) Holland and Co-Worker’s Synthesis of a β-Diketiminate Fe­(II)-Based Macrocycle via C–N Bond Cleavage; (c) Hypothesized Synthesis of Iron-Containing Metallocycles by N–H Activation of Amides Bearing Coordinating Atoms

We were keen to investigate if Fe­(II) β-diketiminate complexes, given the right choice of coligand, could be translated into the formation of metallomacrocyclic species. A sole example of such a species was presented by Holland and co-workers in 2017 (Scheme 1b).^18^ Most examples of macrocyclic species comprised of iron centers reported prior to this were based upon Fe­(II)/Fe­(III) cations bound by neutral bi/terpyridyl moieties or extended architectures comprising elaborate tridentate or higher denticity anionic ligands.^19–29^ However, Holland and co-workers showed that the reaction of an Fe­(I) β-diketiminate species with 4-(dimethylamino)­pyridine (DMAP) resulted in the activation of the dimethylamino moiety to yield two products: (i) a monomeric Fe­(II)–NMe_2_ species bearing a bound DMAP molecule and (ii) a tetrametallic Fe­(II) macrocycle bridged by four pyridyl ligands. Despite this interesting result, no further investigations were undertaken into either the synthesis of further metallomacrocyclic analogs or the potential applications of these species. Here, we have a unique opportunity to study the formation of iron-based metallomacrocycles using simple bidentate ligands and to study onward applications.

## Results and Discussion

Iron­(II) β-diketiminate complex **Fe-1** ^30^ has been shown to activate the N–H bonds of primary and secondary amines, releasing SiMe_4_ and forming new Fe–N bonds.^31,32^ We hypothesized that if an amine bearing a substituent with a coordinating atom was reacted with **Fe-1**, this may lead to bridging coordination between iron centers and subsequent oligomerization in the solid state (Scheme 1c). Indeed, as part of studies into depolymerization catalysis, we recently reported that the reaction of **Fe-1** with morpholine results in the formation of hexamer **Fe-3** in the solid state (Figure 1).^33^ We were therefore keen to investigate how the use of different ligand environments (both amine and β-diketiminate) around the iron centers would affect the formation and ring size of any macrocyclic species. In addition, we wanted to investigate applications of the species on the basis of their properties in the solid state.

**Figure 1:**
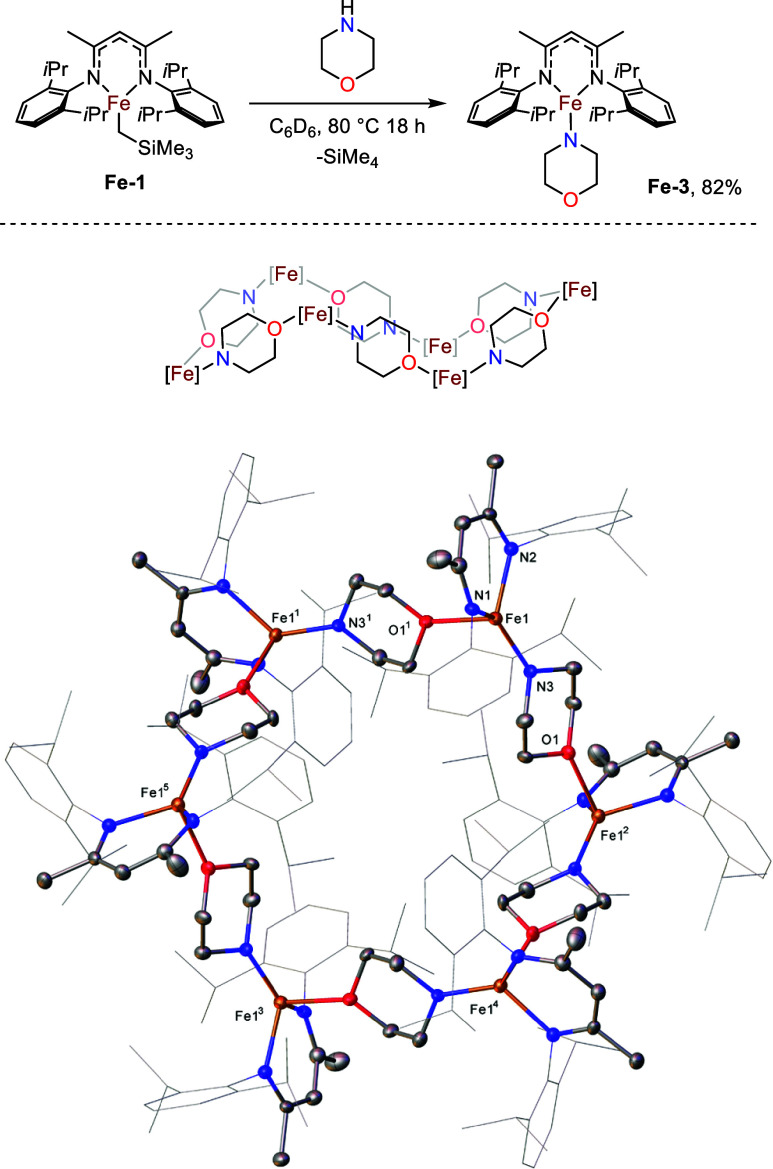
Molecular structure of **Fe-3** (CCDC 1962467). Ellipsoids are represented at a 30% probability. Hydrogen atoms have been omitted, and the β-diketiminate substituents have been represented in wireframe view, for clarity. Symmetry operations^1^:1–*z*, 1–*x*, – *y* ^2^;1–*y*, – *z*, 1–*x* ^3^;2–*x*, – *y*, – *z* ^4^;1+*z*, – 1+*x*, *y* ^5^;1+*y*, *z*, – 1+*x*.

### Synthesis of Metallomacrocyclic (β-Diketiminato)­iron-amides: Reactions with Morpholine and Derivatives

In complex **Fe-3** the morpholine units bridge the iron centers *via* dative interactions with the oxygen atoms at alternating Fe–N and Fe–O bond distances of 1.894(3) and 2.249(3) Å respectively. The iron atoms are arranged such that the hexamer sits in a cyclohexane-like chair conformation, comparable to recently reported metallomacrocyclic structures consisting of pyridyl-bridged magnesium and aluminum metal centers bearing β-diketiminate and diimine ligands respectively.^34,35^ The coordination geometry about the iron centers shows distortions from both trigonal pyramidal and tetrahedral geometries; the three N–Fe–O angles around each iron center have a mean of 101.26°. The cyclic iron tetramer with bridging pyridyl ligands reported by Holland and co-workers showed a distortion toward trigonal pyramidal geometry in the solid state, causing a loss of symmetry between the iron centers due to the alternation between C and N atoms in the axial positions.^18^ In contrast to this, the iron centers of **Fe-3** are equivalent in the solid state, exhibiting a 6-fold pseudosymmetry axis with the morpholine O atoms situated at each of the axial positions of the iron atoms. The angles between adjacent iron centers are therefore all equal. The ^1^H NMR spectrum of **Fe-3** is surprisingly simple and subsequent DOSY analysis of **Fe-3** indicates that the hexamer breaks into monomers in solution,^33^ which is not surprising given the fairly weak dative bonding interaction that holds the cyclic structure together.

Moving forward from this result, we investigated the effect of different bridging amide ligands on the macrocycle formation. We first looked toward derivatives of morpholine to investigate how changing the nature of the coordinating atom would affect the capacity of the species to act as a bridging ligand. The reaction of thiomorpholine with **Fe-1** was therefore undertaken. Again, complete conversion of **Fe-1** to new paramagnetic signals is observed by ^1^H NMR spectroscopy after 18 h at 80 °C. The crude product mixture was crystallized in a toluene/pentane mixture and single crystal X-ray diffraction (SC-XRD) analysis shows the formation of iron amide product **Fe-4** (Figure 2a). However, the structure is monomeric in the solid state, with no coordination to iron via the sulfur atom. An analogous reaction using *N*-methylpiperazine under the same conditions, also revealed the solid-state structure of the resulting iron amide **Fe-5** to be monomeric (Figure 2b). Increasing the number of equivalents of *N*-methylpiperazine simply results in an additional, datively bound, piperazine unit (see the Supporting Information). While it is surprising that the subtle changes in amide substituent from **Fe-3** to **Fe-4** and **Fe-5** shuts down macrocycle formation entirely, this proves that the cyclization occurs under very specific ligand environments and is highly sensitive to the electronic nature and steric environment of the datively bonded atom.

**Figure 2:**
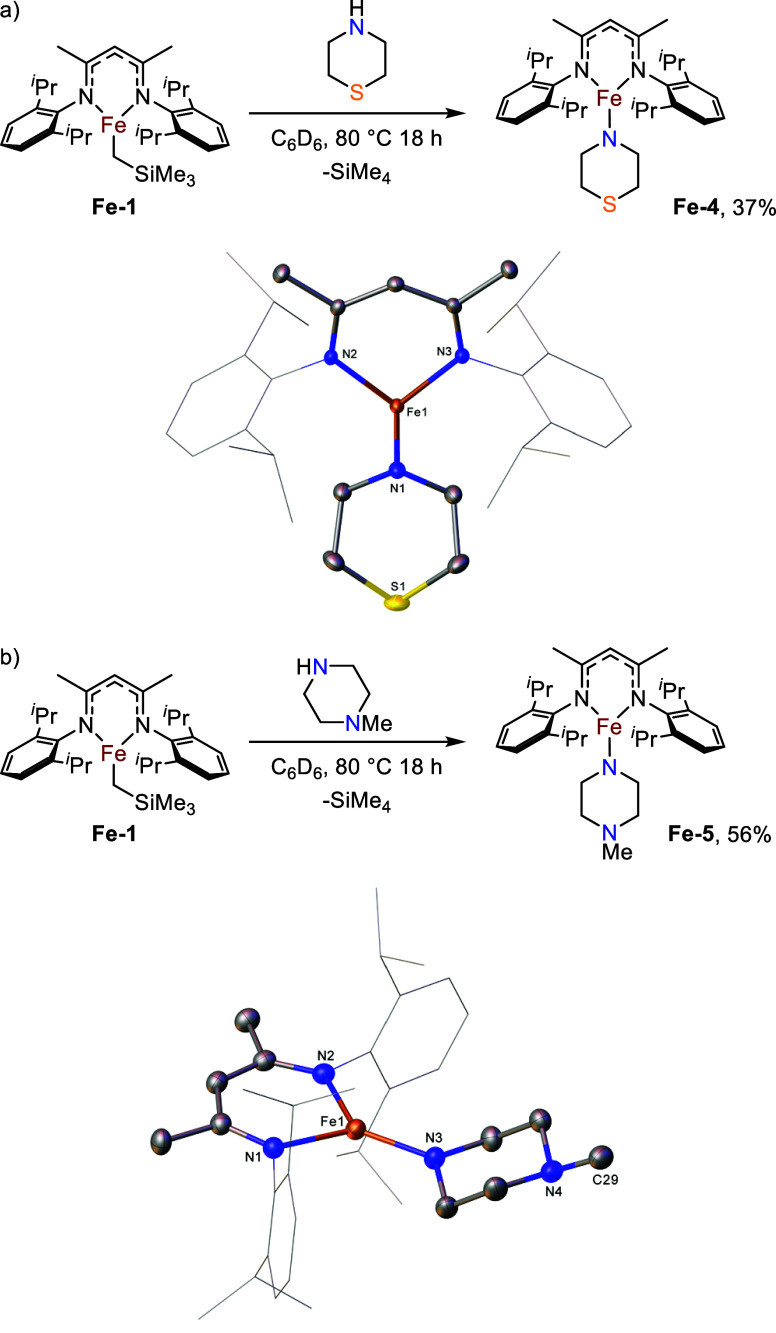
a) Formation of monomeric iron β-diketiminate amide species **Fe-4** and molecular structure of **Fe-4** (CCDC 2211232). Ellipsoids are represented at 30% probability. Hydrogen atoms have been omitted, and the β-diketiminate substituents have been represented in wireframe view, for clarity; b) Formation of monomeric iron β-diketiminate amide species **Fe-5** and the molecular structure of **Fe-5** (CCDC 2211229). Ellipsoids are represented at 30% probability. Hydrogen atoms have been omitted and the β-diketiminate substituents have been represented in wireframe view, for clarity.

### Reactions with Pyridyl-Containing Amines

We next decided to investigate the use of amines bearing pyridyl substituents, given their precedence for use as bridging ligands in macrocyclic structures.^18,34,35^ The reaction of **Fe-1** with 4-aminopyridine leads to complete consumption of **Fe-1** after 1 h at 80 °C. **Fe-6** crystallizes as a cyclic tetramer in 61% yield, with iron centers bridged by dative bonds from the pyridyl moieties to adjacent iron centers (Figure 3). Contrary to the tetramer reported by Holland and co-workers, the iron centers in **Fe-6** are skewed rather than exhibiting a planar distribution because of the presence of the amido functionality in **Fe-6**. The distances and angles between the iron centers are therefore inequivalent. The iron amide bonds of **Fe-4** have a considerably longer mean distance of 1.962 Å in comparison to the Fe–N bonds of **Fe-3** (1.894(3) Å). The average pyridyl N–Fe dative interaction distance (Fe–N = 2.078 Å) is comparatively shorter than the analogous Fe–O interaction in **Fe-3** (Fe–O = 2.249(3) Å). The mean angle between the formal Fe–N covalent bonds and the dative Fe–N interactions in **Fe-6** is 107.37°; therefore, the iron centers here are best described as having a distorted tetrahedral geometry.

**Figure 3:**
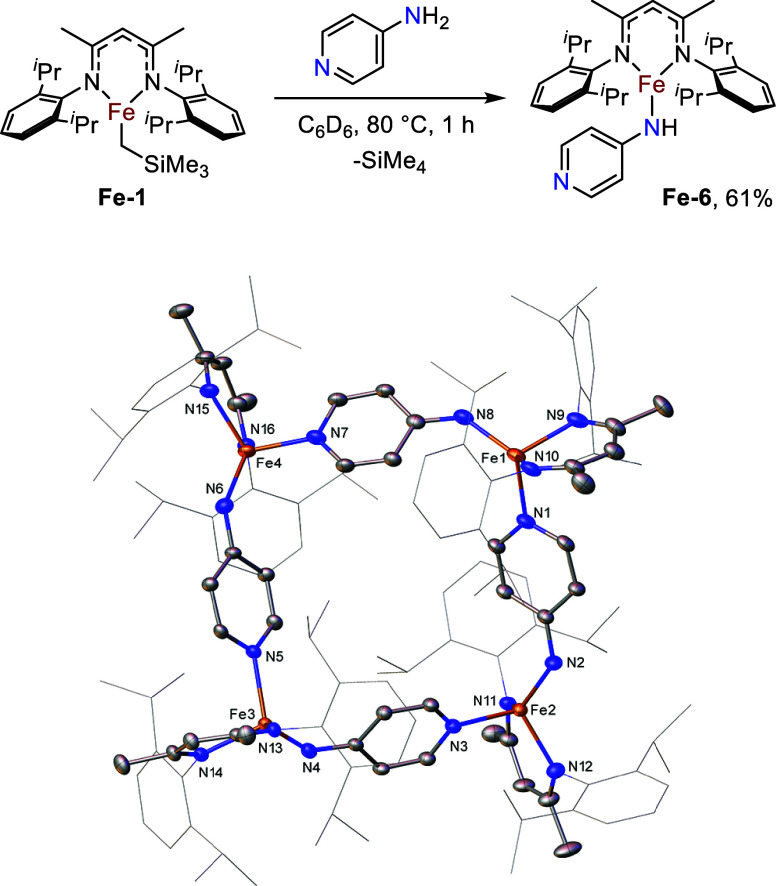
Synthesis of tetrameric iron β-diketiminate amide species **Fe-6** and the molecular structure of **Fe-6**. Ellipsoids are represented at 30% probability. Hydrogen atoms have been omitted, and the β-diketiminate substituents have been represented in wireframe view, for clarity (CCDC 2211233).

This is reflected in the ^1^H NMR spectrum of **Fe-6**, which contains a complex mixture of signals in the paramagnetic window. The paramagnetic nature of the complex means that DOSY analysis has been challenging and somewhat ambiguous, but the complexity of the ^1^H NMR spectrum implies that the tetrameric structure or mixed species are present in solution to some extent. If there was complete decomposition into monomers, we would anticipate a simplified NMR spectrum.

We next investigated the effects of changing the position of the pyridyl coordinating atom of the bridging amine ligand. The reaction of 3-aminopyridine with **Fe-1** leads to the formation of an insoluble brown precipitate, and despite multiple crystallization efforts, only amorphous material was obtained and investigations into the reaction were not pursued further. Success was found with 2-aminopyridine; addition of the amine to **Fe-1** gives an instant red color change, followed by full consumption of **Fe-1** by ^1^H NMR spectroscopy after one hour at 80 °C. Crystallization of the resulting crude red solid from toluene at – 30 °C yields red crystals after 18 h. Analysis by SC-XRD revealed the asymmetric structure to be the iron amide species **Fe-7** (Figure 4). The asymmetric unit of **Fe-7** constitutes half of a pyridyl-bridged dimer, with the reduction in metallomacrocycle size from **Fe-6** correlating with the reduction in the angle between the amine and pyridine moieties of the two species.

**Figure 4:**
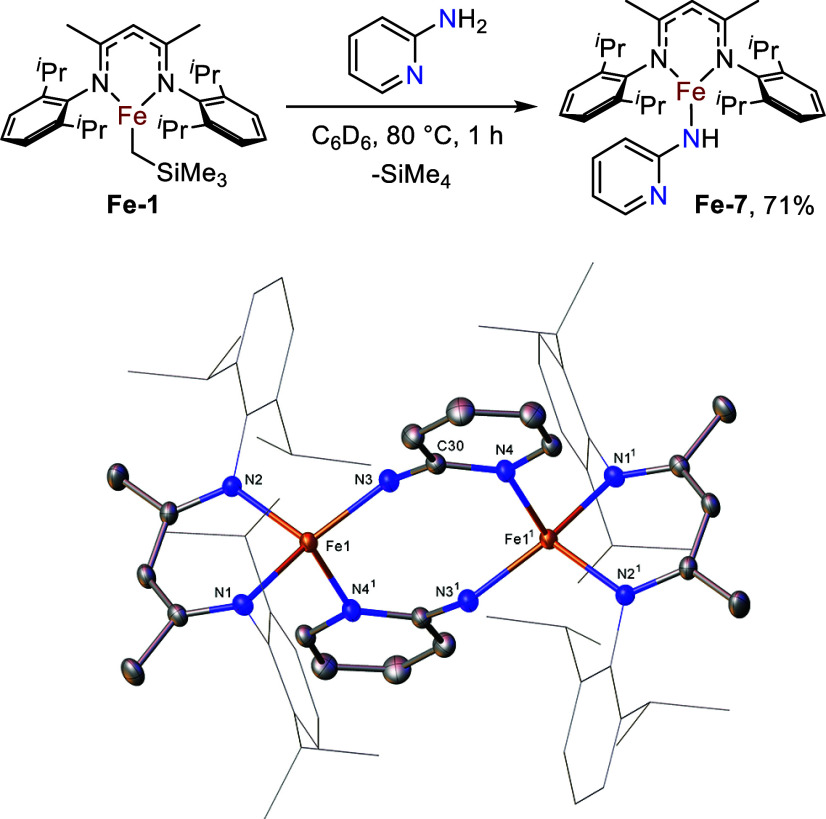
Synthesis and Molecular structure of **Fe-7**. Ellipsoids are represented at 30% probability. Both solvent and hydrogen atoms have been omitted, and the β-diketiminate substituents have been represented in wireframe view, for clarity. Symmetry operations^1^:1–*x*, 1–*y*, 1–*z*. (CCDC 2211234).

We next looked to extend the distance between the amine and pyridyl components of the bridging ligand and investigate any effect this would have on the formation of Fe–Fe linkages. A reaction between **Fe-1** and *N*-methylpyridine-4-methylamine results in iron amide **Fe-8** (Figure 5), which exhibits a trimeric structure with bridging Fe-pyridyl dative interactions in the solid state. In comparison to the bridging 4-aminopyridine ligand in **Fe-6**, where the pyridyl-N and amine moieties sit in the plane of the pyridyl ring, the methylene group between the amine and pyridyl environments in *N*-methylpyridine-4-methylamine results in the loss of this planarity in **Fe-8**. Subsequently, the iron centers lie at tighter angles than one another, resulting in a trimeric metallomacrocycle rather than the tetramer observed for **Fe-6**. Similar to **Fe-6**, **Fe-8** can be heated under vacuum and the ^1^H NMR spectrum is retained on dissolution in C_6_D_6_.

**Figure 5:**
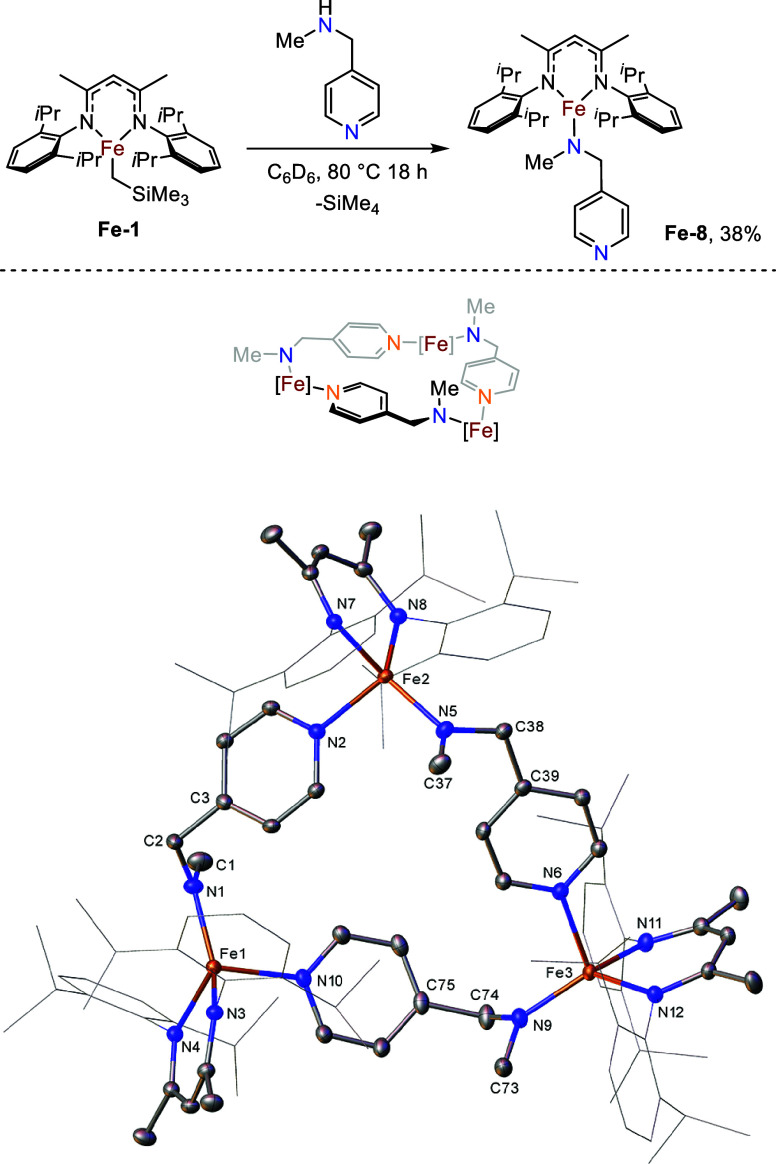
Synthesis and molecular structure of **Fe-8** (CCDC 2211235). Ellipsoids are represented at 30% probability. Both solvent and hydrogen atoms have been omitted and the β-diketiminate substituents have been represented in wireframe view, for clarity.

Finally, 2-picolylamine was reacted with **Fe-1**. After a deep red color change on addition of the amine, complete consumption of signals corresponding to **Fe-1** is observed by ^1^H NMR spectroscopy after a further hour at 80 °C. Crystallizing the crude material from pentane at – 30 °C yields deep red crystals. SC-XRD analysis reveals that the methylene alters the coordination mode of the pyridyl group such that chelation about a single iron center is preferential to bridging between iron centers (Figure 6). **Fe-9** possesses Fe-amide and Fe-pyridyl distances of similar lengths to the complexes previously discussed (Fe–N1 = 1.929(4) Å, Fe–N2 = 2.111(4) Å). The bite angle of the chelating ligand is tighter than the respective angles between the Fe-amide bond and the bridging dative bonds in structures **Fe-3**, **Fe-6**, **Fe-7**, and **Fe-8** however (**Fe-9**: N1–Fe–N2 = 81.5(1)°, **Fe-3**: N1–Fe–O’ = 101.7(1), **Fe-6**: N1–Fe–N2’ (mean) = 111.4°, **Fe-7**: N1–Fe–N2’ (mean) = 105.8°, **Fe-8**: N1–Fe–N2’ = 99.3(6)°).

**Figure 6:**
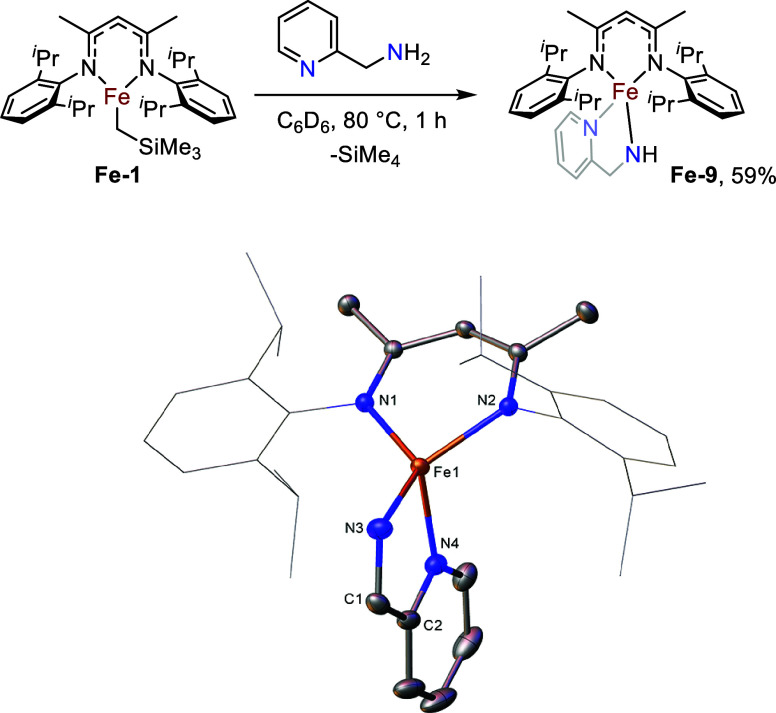
Synthesis and molecular structure of **Fe-9**. Ellipsoids are represented at 30% probability. Hydrogen atoms have been omitted, and the β-diketiminate substituents have been represented in wireframe view, for clarity. (CCDC 2211236).

### β-Diketiminate Variants

We next decided to investigate how variation of β-diketiminate would affect macrocyclization. An analog of **Fe-1** bearing a 2,6-dimethylphenyl substituted β-diketiminate ligand (**Fe-2**) was synthesized according to a literature procedure.^30^ **Fe-2** bears less steric bulk around the iron center in comparison to **Fe-1** due to the smaller flanking aryl groups, which we hypothesized may lead to more freedom in coordination for the bridging ligand and consequently to the formation of new variations of metallomacrocycle.

Addition of morpholine and *N*-methylpiperazine to **Fe-2** results in an instant red color change in each case, shortly followed by red crystals dropping out of both solutions. Analysis of the crystals by SC-XRD reveals that in both cases a dimer had formed, with the iron centers bridged by two three-center-two-electron bonds involving the N-atom of the resulting amide (Figure 7, **Fe-10** and **Fe-11**). This reinforces how seemingly small differences to ligand structure can lead to large differences in coordination chemistry.

**Figure 7:**
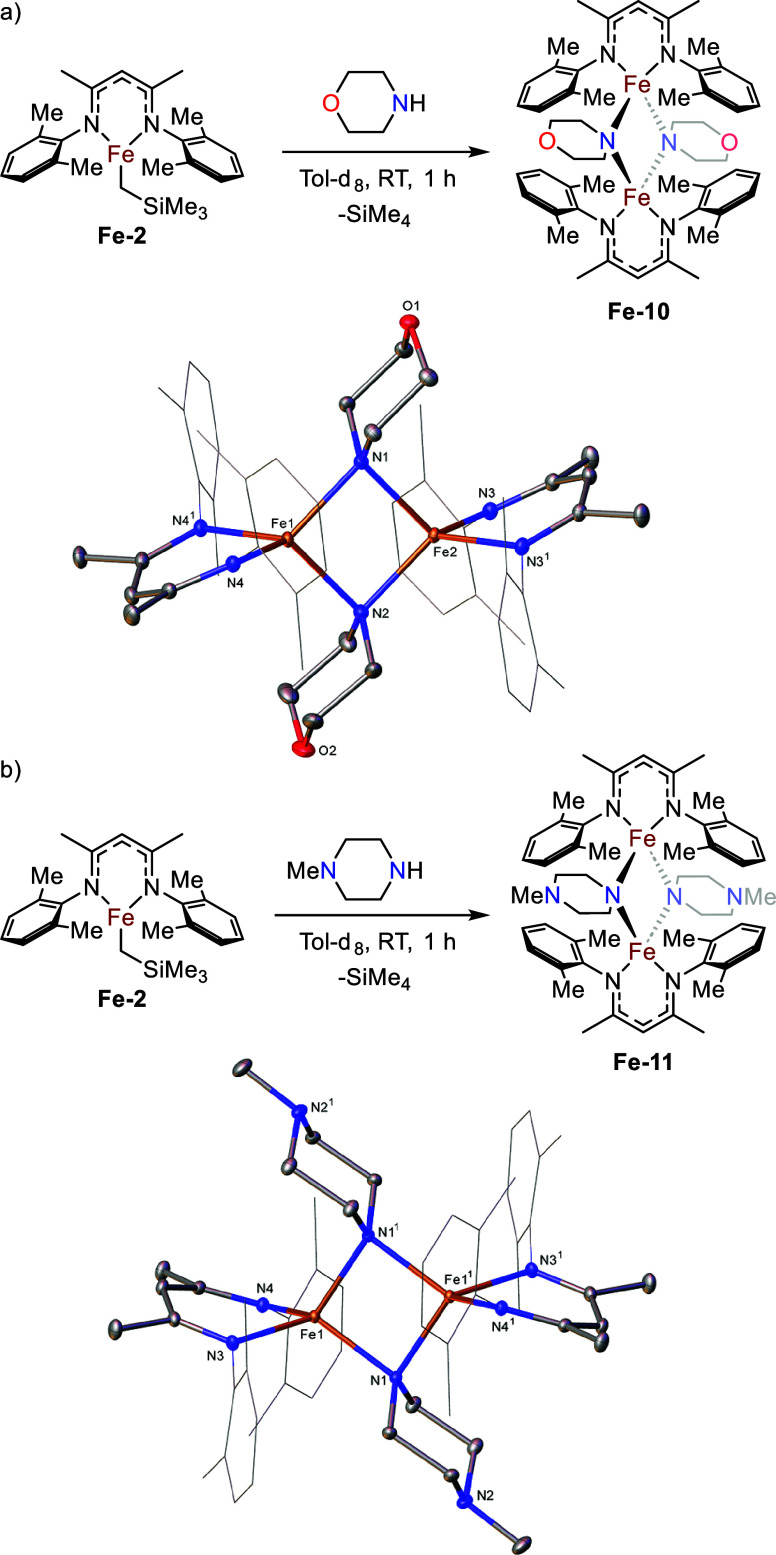
a) Synthesis of dimeric iron amide **Fe-10** and molecular structure of **Fe-10** (CCDC 2211228). Ellipsoids are represented at 30% probability. Both solvent and hydrogen atoms have been omitted and the β-diketiminate substituents have been represented in wireframe view, for clarity. Symmetry operations: ^1^ *x*, 1/2–*y*, *z*; b) Synthesis of dimeric iron amide **Fe-11** and one of the two independent molecules present in the structure of **Fe-11** (CCDC 2211230). Ellipsoids are represented at 30% probability. Hydrogen atoms have been omitted and the β-diketiminate substituents have been represented in wireframe view, for clarity. Symmetry operations^1^:1–*x*, 1–*y*, 1–*z*.

A comparison of Fe–Fe distances and angles for the metallomacrocyclic species synthesized is presented in Table 1 and the outcome of the coordination chemistry studies is summarized in Figure 8.

**Table 1:** Comparisons of key bond metrics in metallomacrocyclic systems **Fe-3**, **Fe-6**, and **Fe-8**

Species	Fe–(N–E)–Fe, Å	Fe–Fe–Fe, °	Fe–N, Å	Fe–E, Å
**Fe-3**	7.009	93.62(2)	1.894(3)	2.249(3)
**Fe-6**	7.7809^a^	85.28^a^	1.962^a^	2.078^a^
**Fe-8**	8.4175^a^	60.6^a^	1.916^a^	2.131^a^

a Mean of all relevant metrics in structure.

**Figure 8:**
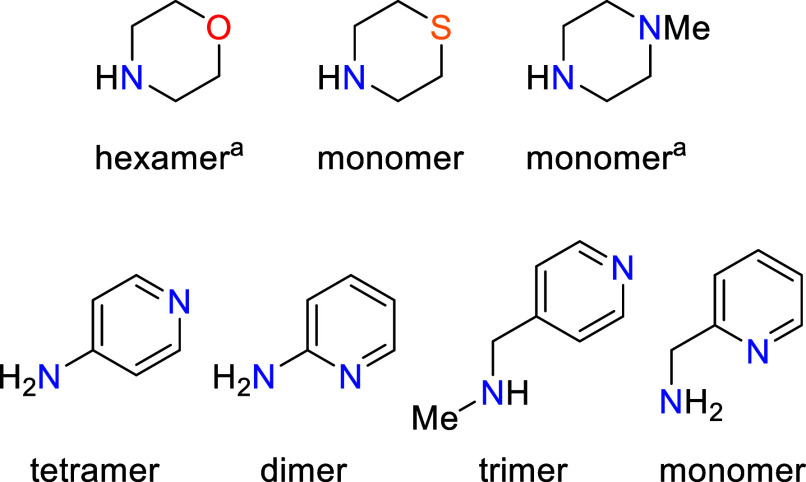
An overview of the coordination environment for different amide linkers tested. ^a^Dimer forms when **Fe-2** is employed as the precursor.

### Solid-State Analysis and Applications of **Fe-3**, **Fe-6**, and **Fe-8**

Packing analysis of **Fe-3**, **Fe-6**, and **Fe-8** show extended structures in the solid state and **Fe-3**, in particular, is somewhat reminiscent of a MOF-type species (Figure 9). We therefore hypothesized that if the residual solvent present in the unit cells of **Fe-3**, **Fe-6**, and **Fe-8** could be removed while maintaining the structural integrity of the unit cells, then porosity and thus adsorption of gases would be viable.

**Figure 9:**
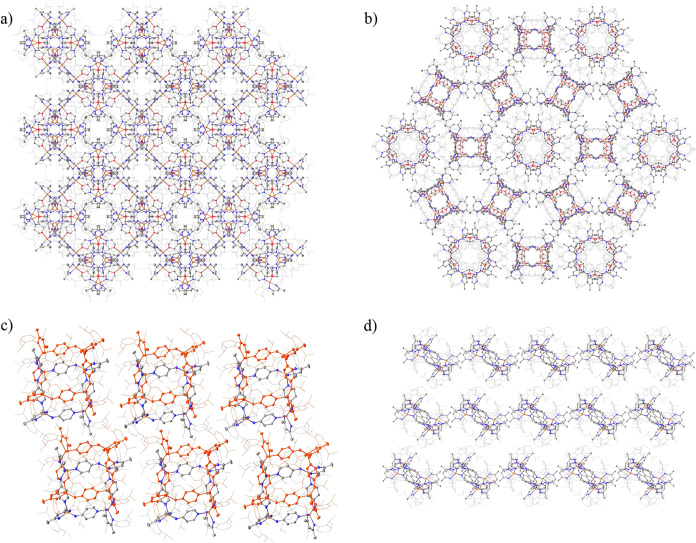
a) The gross structure of **Fe-3** (solvent omitted) viewed along the [001] direction and (b) the [111] direction, emphasizing the accessible channels in the array. Hydrogen atoms have been omitted and the β-diketiminate substituents have been represented in wireframe view, for clarity. c) Gross structure of **Fe-6** (solvent omitted) viewed along the [010] direction showing the offset layers of molecules that stack along the *b*-axis. Hydrogen atoms have been omitted and the β-diketiminate substituents have been represented in wireframe view, for clarity. d) The gross structure of **Fe-8** (solvent omitted) viewed along the [011] direction emphasizing the accessible channels in the array. Hydrogen atoms have been omitted and the β-diketiminate substituents have been represented in wireframe view, for clarity.

To investigate this the ‘solvent accessible’ surface percentage was calculated for the unit cell of **Fe-3**, **Fe-6** and **Fe-8** to probe the potential for these complexes to adsorb gases such as CO_2_ and N_2_.^36,37^ A general trend is that the smaller the macrocycle size, the lower the volume of the unit cell is predicted to be accessible by both gases. For CO_2_, the theoretical adsorption volume is 5.9% for **Fe-3**, 4.3% for **Fe-6** and 3.9% for **Fe-8**. For N_2_, the theoretical adsorption volume is 4.6% for **Fe-3**, 2.9% for **Fe-6** and 2.3% for **Fe-8**. Initially the porosity, pore structure, and surface area of **Fe-3** was analyzed using CO_2_. Unfortunately, **Fe-3** shows abnormal behavior under an atmosphere of CO_2,_ with the sample pressure failing to stabilize. This suggests that rather than diffusing through the sample, the CO_2_ may have bonded directly to the outer layer of **Fe-3** and, subsequently, any adsorption data could not be accurately obtained. Qualitatively we observe a color change when the solid is placed under an atmosphere of CO_2_, indicating a change in coordination at the metal center. This competitive binding of the Lewis basic CO_2_ lone pairs with an iron center is in line with studies on Lewis basic binding from Guedes da Silva and Pombeiro, where substituted pyridines and imidazole were observed to bind to iron in a metallomacrocycle.^38^

We therefore moved on to investigate the adsorptive properties of **Fe-3**, **Fe-6**, and **Fe-8** with N_2_. Pleasingly, the analysis of **Fe-3** using N_2_ was successful, and a physisorption type II isotherm, typical of a moderately porous sample, is observed (Figure 10). The surface area of **Fe-3** (calculated using the Brunauer–Emmett–Teller (BET) model)^39^ is 20.2 m^2^ g^–1^, and the pore size distribution (calculated using the Barrett–Joyner–Halenda model)^40^ implies a cylindrical pore size of 3 to 4 nm, in line with **Fe-3** being a mesoporous material. **Fe-6** is similar to a surface area of 21.1 m^2^ g^–1^, but with a broader pore size distribution (2 to 5 nm). In contrast, the reduction in ring size is detrimental for the trimer **Fe-8**, which has a much-reduced surface area of 8.2 m^2^ g^–1^. However, **Fe-8** has a pore size distribution comparable to that of the tetramer (2 to 5 nm), indicating that it is still mesoporous in nature.

**Figure 10:**
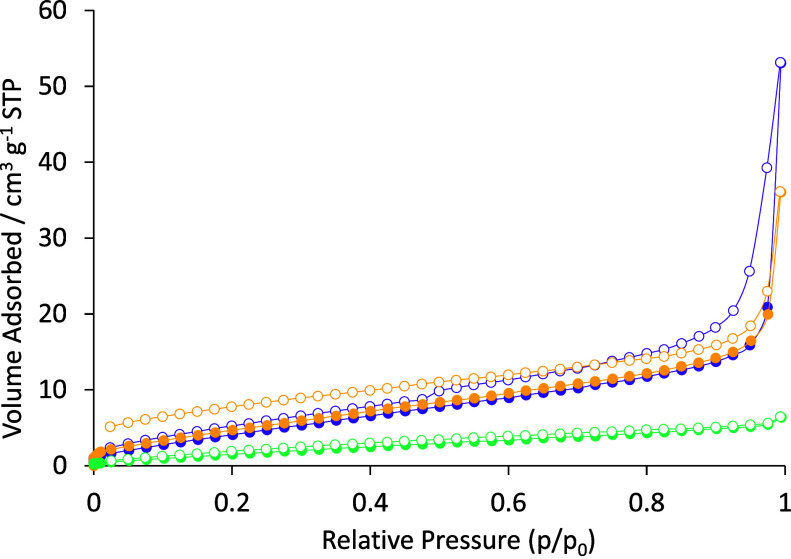
Isotherms showing the adsorption and desorption of N_2_ gas by **Fe-3** (●/○; purple closed and open circle),**Fe-6** (●/○; yellow closed and open circle) and **Fe-8** (●/○; turquoise closed and open circle). Analysis was undertaken at 77 K.

## Conclusions

This represents an initial foray into the potential application of iron metallomacrocycles as mesoporous gas adsorbents. Three macrocyclic species, involving 2,6-diisopropyl substituted β-diketiminate-bound iron centers, were synthesized, isolated, and structurally characterized by SC-XRD. The use of amide ligands bearing an additional coordinating atom was imperative to the formation of these macrocyclic species, allowing for the formation of a dative bond to an adjacent iron center to bridge the metal complexes. Varying the amide ligand resulted in the formation of macrocycles consisting of three (amide = *N*-methylpyridine-4-methylamine, **Fe-8**), four (amide = 4-aminopyridine, **Fe-6**) and six (amide = morpholine, **Fe-3**) iron centers. Attempts to synthesize further macrocyclic derivatives proved difficult, with morpholine derivatives (*N*-methylpiperazine and thiomorpholine) providing monomeric structures in the solid state. Use of amide ligands with a tighter angle between the amine and the coordinative moiety resulted either in chelation about the iron center (2-picolylamine) or dimerization (2-aminopyridine). Investigations into metallomacrocycle formation with the less sterically encumbered 2,6-dimethylphenyl-β-diketiminate-bound iron species were hindered by the preferential formation of Fe–N–Fe bridged dimeric species.

Theoretical investigations into the porosity of the unit cells revealed that the macrocyclic species may have accessible pores in the solid state. While analysis using CO_2_ was found to be unfeasible, preliminary experimental analysis of **Fe-3**, **Fe-6**, and **Fe-8** revealed a N_2_ physisorption isotherm synonymous with mesoporous samples, matching the theoretical predictions.

## Supplementary Material



## Notes

Synthesis and SC-XRD data (PDF)

## ‡

Present address: Henry Royce Institute, University of Manchester, Manchester, M13 9PL, England (R.C.)

## References

[1] Fujita M., Yazaki J., Ogura K. (1990). Preparation of a macrocyclic polynuclear
complex, [(en)­Pd­(4,4’-bpy)]_4_(NO_3_)_8_ (en = ethylenediamine, bpy = bipyridine), which recognizes
an organic molecule in aqueous media. J. Am.
Chem. Soc..

[2] Stang P. J., Cao D. H. (1994). Transition Metal Based Cationic Molecular Boxes. Self-Assembly
of Macrocyclic Platinum­(II) and Palladium­(II) Tetranuclear Complexes. J. Am. Chem. Soc..

[3] Jones C. (1998). Transition
metals as structural components in the construction of molecular containers. Chem. Soc. Rev..

[4] Chakrabarty R., Mukherjee P. S., Stang P. J. (2011). Supramolecular Coordination: Self-Assembly
of Finite Two- and Three-Dimensional Ensembles. Chem. Rev..

[5] Fujita M., Nagao S., Iida M., Ogata K., Ogura K. (1993). Palladium­(II)-directed
assembly of macrocyclic dinuclear complexes composed of (en)­Pd^2+^ and bis­(4-pyridyl)-substituted bidentate ligands. Remarkable
ability for molecular recognition of electron-rich aromatic guests. J. Am. Chem. Soc..

[6] Stang P. J., Cao D. H., Saito S., Arif A. M. (1995). Self-Assembly of
Cationic, Tetranuclear, Pt­(II) and Pd­(II) Macrocyclic Squares. x-ray
Crystal Structure of [Pt^2+^(dppp)­(4,4’-bipyridyl)·2-OSO_2_CF_3_]_4_. J. Am.
Chem. Soc..

[7] Severin K. (2003). Self-assembled
organometallic receptors for small ions. Coord.
Chem. Rev..

[8] Vajpayee V., Kim H., Mishra A., Mukherjee P. S., Stang P. J., Lee M. H., Kim H. K., Chi K.-W. (2011). Self-assembled
molecular squares
containing metal-based donor: synthesis and application in the sensing
of nitro-aromatics. Dalton Trans..

[9] Vajpayee V., Song Y. H., Lee M. H., Kim H., Wang M., Stang P. J., Chi K. W. (2011). Self-Assembled Arene–Ruthenium-Based
Rectangles for the Selective Sensing of Multi-Carboxylate Anions. Chem.—Eur. J..

[10] Walter C. J., Anderson H. L., Sanders J. K. M. (1993). exo-Selective acceleration of an
intermolecular Diels–Alder reaction by a trimeric porphyrin
host. J. Chem. Soc. Chem. Commun..

[11] Oliveri C. G., Gianneschi N. C., Nguyen S. T., Mirkin C. A., Stern C. L., Wawrzak Z., Pink M. (2006). Supramolecular Allosteric Cofacial
Porphyrin Complexes. J. Am. Chem. Soc..

[12] Oliveri C.
G., Heo J., Nguyen S. T., Mirkin C. A., Wawrzak Z. (2007). A Convergent Coordination
Chemistry-Based Approach to Dissymmetric Macrocyclic Cofacial Porphyrin
Complexes. Inorg. Chem..

[13] Gianneschi N. C., Cho S.-H., Nguyen S. T., Mirkin C. A. (2004). Reversibly Addressing
an Allosteric Catalyst In Situ: Catalytic Molecular Tweezers. Angew. Chem., Int. Ed..

[14] Cotton F. A., Murillo C. A., Stiriba S.-E., Wang X., Yu R. (2005). Chiral Organometallic
Triangles with Rh–Rh Bonds. 2. Compounds Prepared from Enantiopure *cis*-Rh_2_(C_6_H_4_PPh_2_)_2_(OAc)_2_(HOAc)_2_ and Their Catalytic
Potentials. Inorg. Chem..

[15] Mattsson J., Govindaswamy P., Renfrew A. K., Dyson P. J., Štěpnička P., Süss-Fink G., Therrien B. (2009). Synthesis, Molecular Structure, and
Anticancer Activity of Cationic Arene Ruthenium Metallarectangles. Organometallics.

[16] Barry N. P. E., Edafe F., Dyson P. J., Therrien B. (2010). Anticancer activity
of osmium metalla-rectangles. Dalton Trans..

[17] Barry N. P. E., Edafe F., Therrien B. (2011). Anticancer
activity of tetracationic
arene ruthenium metalla-cycles. Dalton Trans..

[18] Macleod K. C., Lewis R. A., Derosha D. E., Mercado B. Q., Holland P. L. (2017). C–H
and C–N Activation at Redox-Active Pyridine Complexes of Iron. Angew. Chem., Int. Ed..

[19] Burkill H. A., Robertson N., Vilar R., White A. J. P., Williams D. J. (2005). Synthesis,
Structural Characterization, and Magnetic Studies of Polynuclear Iron
Complexes with a New Disubstituted Pyridine Ligand. Inorg. Chem..

[20] Constable E. C., Dunphy E. L., Housecroft C. E., Kylberg W., Neuburger M., Schaffner S., Schofield E. R., Smith C. B. (2006). Structural Development
of Free or Coordinated 4′-(4-Pyridyl)-2,2′:6′,2″-terpyridine
Ligands through N-Alkylation: New Strategies for Metallamacrocycle
Formation. Chem.—Eur. J..

[21] Jiang T.-Y., Zhong H.-J., Xiang T., Jin L.-F., Yu H. (2009). Synthesis,
crystal structure and esterification of a novel iron­(III) 18-metallacrown-6
complex. Inorg. Chim. Acta.

[22] Jin L., Yu H., Wu S., Xiao F. (2009). Iron­(iii) coordination induced novel
18-metallacrown-6 complex: esterification and isolation of the ligand. Dalton Trans..

[23] Shu T.-P., Wen J.-L., Feng H.-M., Lei K.-W., Liang H.-Z. (2009). Synthesis,
structural characterization and magnetic properties of a novel metallacrown
[Fe_6_(amshz)_6_(C_3_H_7_NO)_6_]·6CH_3_OH. Solid State
Sci..

[24] Lei K.-W., Feng H.-M., Wen J.-L., Shu T.-P. (2010). Synthesis, structural
characterization, and magnetic properties of the metallamacrocycle
[Fe_6_(C_11_H_11_N_2_O_3_)_6_(C_4_H_9_NO)_6_]. Monatsh. Chem..

[25] Jin C., Yu H., Jin L., Wu L., Zhou Z. (2010). Esterification and
isolation of the carboxylic acid with salicyl-bis-hydrazide via coordination
of iron­(III) 18-metallacrown-6 complex. J. Coord.
Chem..

[26] Jin C.-Z., Wu S.-X., Jin L.-F., Wu L.-M., Zhang J. (2012). Esterification
of the ligand: Synthesis, characterization and crystal structure of
a iron­(III) 18-metallacrown-6 complex with methyl 4-(5′-chlorosalicylhydrazinocarbonyl)
butyrate. Inorg. Chim. Acta.

[27] Hasenknopf B., Lehn J.-M., Kneisel B. O., Baum G., Fenske D. (1996). Self-Assembly
of a Circular Double Helicate. Angew. Chem.,
Int. Ed..

[28] Hasenknopf B., Lehn J.-M., Boumediene N., Dupont-Gervais A., Van Dorsselaer A., Kneisel B., Fenske D. (1997). Self-Assembly of Tetra-
and Hexanuclear Circular Helicates. J. Am. Chem.
Soc..

[29] Hwang S.-H., Moorefield C. N., Fronczek F. R., Lukoyanova O., Echegoyen L., Newkome G. R. (2005). Construction of triangular metallomacrocycles:
[M_3_(1,2-bis­(2,2′ : 6′,2″-terpyridin-4-yl-ethynyl)­benzene)_3_] [M = Ru^(ii)^, Fe^(ii)^, 2Ru^(ii)^Fe^(ii)^]. Chem. Commun..

[30] Sciarone T. J. J., Meetsma A., Hessen B. (2006). Neutral and
cationic Fe­(II) β-diketiminate
complexes. Inorg. Chim. Acta.

[31] Gasperini D., King A., Coles N. T., Mahon M. F., Webster R. L. (2020). Seeking
Heteroatom-Rich Compounds: Synthetic and Mechanistic Studies into
Iron Catalyzed Dehydrocoupling of Silanes. ACS
Catal..

[32] Bernoud E., Oulié P., Guillot R., Mellah M., Hannedouche J. (2014). Well-Defined
Four-Coordinate Iron­(II) Complexes For Intramolecular Hydroamination
of Primary Aliphatic Alkenylamines. Angew. Chem.,
Int. Ed..

[33] Farcaş-Johnson M. A., Gasperini D., King A. K., Mohan S., Barrett A. N., Lau S., Mahon M. F., Sarazin Y., Kyne S. H., Webster R. L. (2023). Iron­(II)-Catalyzed
Activation of Si–N and Si–O Bonds Using Hydroboranes. Organometallics.

[34] Chen W., Liu L., Zhao Y., Xue Y., Xu W., Li N., Wu B., Yang X.-J. (2021). Organometallo-macrocycle
assembled through dialumane-mediated
C–H activation of pyridines. Chem. Commun..

[35] Lawrence S.
R., De Vere-Tucker M., Slawin A. M. Z., Stasch A. (2021). Synthesis of a Hexameric
Magnesium 4-pyridyl Complex with Cyclohexane-like Ring Structure via
Reductive C-N Activation. Molecules.

[36] Aguilar-Armenta G., Patiño-Iglesias M. E., Leyva-Ramos R. (2003). Adsorption
Kinetic Behaviour of Pure CO_2_, N_2_ and CH_4_ in Natural Clinoptilolite at Different Temperatures. Adsorp. Sci. Technol..

[37] Macrae C. F., Bruno I. J., Chisholm J. A., Edgington P. R., McCabe P., Pidcock E., Rodriguez-Monge L., Taylor R., van de Streek J., Wood P. A. (2008). Mercury CSD 2.0
- new features for the visualization and investigation of crystal
structures. J. Appl. Crystallogr..

[38] Sutradhar M., Guedes da Silva M. F.
C., Pombeiro A. J. L. (2013). Synthesis
and
chemical reactivity of an Fe­(III) metallacrown-6 towards N-donor Lewis
bases. Inorg. Chem. Commun..

[39] Brunauer S., Emmett P. H., Teller E. (1938). Adsorption
of Gases in Multimolecular
Layers. J. Am. Chem. Soc..

[40] Barrett E. P., Joyner L. G., Halenda P. P. (1951). The Determination
of Pore Volume
and Area Distributions in Porous Substances. I. Computations from
Nitrogen Isotherms. J. Am. Chem. Soc..

