# Supplementary material for: Structural and Gas Uptake Studies on Iron(II) β‑Diketiminate Metallomacrocycles
Source: Organometallics. 2025 Aug 1;44(15):1713–20. doi: 10.1021/acs.organomet.5c00208 (PMC12344765; doi:10.1021/acs.organomet.5c00208)
Supplement: Supplementary file 1 [file om5c00208_si_001.pdf]

Supporting Information

Structural and Gas Uptake Studies on Iron(II) β-Diketiminate Metallomacrocycles

Adam N. Barrett,[‡a] Leah Webster,[‡b] Danila Gasperini,[a] Rémi Castaing,[c] Mary F. Mahon,[a] Ruth L. Webster[b*]

[a]Department of Chemistry, University of Bath, Claverton Down, Bath, BA2 7AY. United Kingdom
[b]Yusuf Hamied Department of Chemistry, University of Cambridge, Lensfield Road, Cambridge, CB2 1EW. United Kingdom
[c]Chemical Characterisation Facility, Research Infrastructure and Facilities, University of Bath, Claverton Down, Bath, BA2 7AY. United Kingdom

rw740@cam.ac.uk

Contents

1. General Considerations 1
2. Synthetic and Spectroscopic Details of Iron Compounds 2
4. Table S1: Crystallographic Information 10
5. References 13

1. General Considerations

All manipulations were carried out under an inert atmosphere using standard Schlenk and glovebox techniques, unless otherwise stated. Morpholine, *N*-methylpiperazine, thiomorpholine, *N*-methylpyridine-4-methylamine and 2-picolylamine were purchased from commercial sources, dried over calcium chloride, and distilled before use. 4-Aminopyridine and 2-aminopyridine were purchased from commercial sources and sublimed before use. *n*-Butyllithium was purchased from commercial sources and used as supplied. Pinacolborane was purchased from commercial sources and distilled before use. $LiCH_2SiMe_3$ was purchased from a commercial source as a solution in hexanes and was concentrated *in vacuo* to be used in a glovebox as a solid. **Fe-1** and **Fe-2** were synthesised according to literature procedures.[1] Anhydrous C_6D_6 was purchased from Merck and dried over sodium/benzophenone and distilled before use. Pentane, diethyl ether, THF, and toluene were dried over sodium/benzophenone and distilled before use. NMR data was collected at 400 or 500 MHz on Bruker or Agilent instruments in C_6D_6 at 298 K and referenced to residual protic solvent. Satisfactory elemental analysis was not achieved in all cases, likely due to residual crystallisation solvent. Crystal structures were obtained from either a Rigaku Oxford Diffraction Xcalibur (MoKα (λ = 0.71073)) or Supernova (CuKα (λ = 1.54184)) diffractometer. Nitrogen gas adsorption analysis at 77 K was performed with an Autosorb-iQ-C instrument by Quantachrome. BET model (Brunauer-Emmett-Teller)[2] was used to calculate the surface area and BJH model (Barrett-Joyner-Halenda)[3] to calculate the pore size distribution, assuming cylindrical pores.

2. Synthetic and Spectroscopic Details of Iron Compounds

Fe-3:

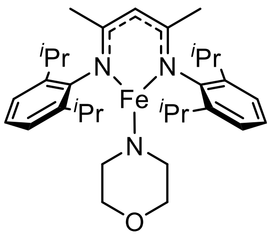

Fe-1 (80 mg, 0.14 mmol) was added to a J-Young NMR tube alongside C_6D_6 (0.6 mL). Morpholine (12.3 µL, 0.14 mmol) was then added and an instant colour change was observed from yellow to red. The reaction was then heated to 80 °C for 18 h. Volatiles were then removed *in vacuo* and the resulting red residue was redissolved in a minimum of pentane. Crystallisation of the solution at -30 °C for three days yielded deep red crystals of **Fe-3**. Upon drying, the crystals undergo a colour change to give **Fe-3** as a brown/green powder (66 mg, 82%).

^{1}H NMR (500 MHz, 298 K, C_6D_6): 108.45 (1H, γ-C*H*), -0.56 (2H, ^{i}Pr-C*H*), -4.74 (8H, morpholine - C*H$_2$*), -11.18 (6H, backbone-C*H$_3$*), -15.86 (2H, ^{i}Pr-C*H*), -20.18 (12H, ^{i}Pr-C*H$_3$*), -81.92 (2H, *para*-Ar-*H*), -102.13 (4H, *meta*-Ar-*H*), -119.03 (12H, ^{i}Pr-C*H$_3$*).

Elemental Analysis: calcd. for $C_{33}H_{49}FeN_3O$: C: 70.83%, H: 8.83%, N: 7.51%; found: C: 70.34, H: 8.90%, N: 7.29%.

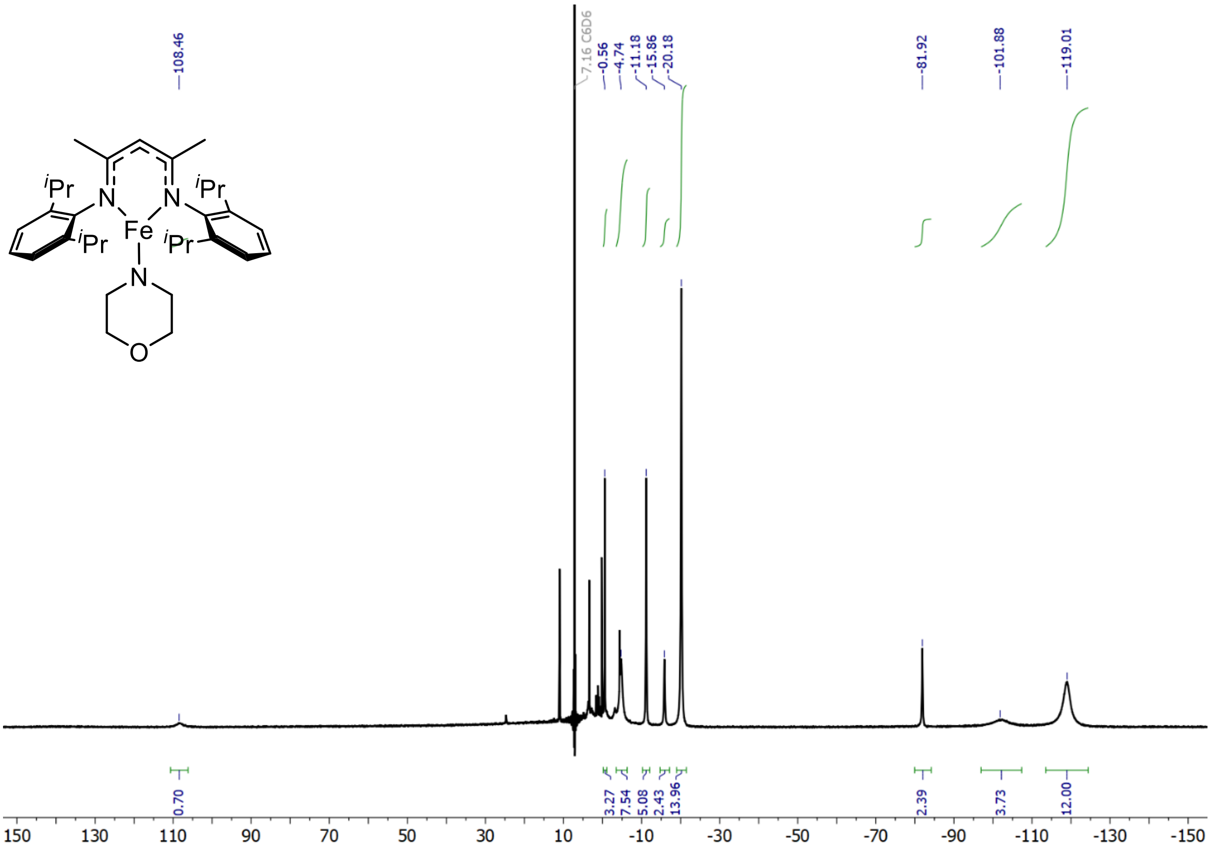

Figure S1: ^{1}H NMR (500 MHz, 298 K, C_6D_6) of **Fe-3.**

Fe-4:

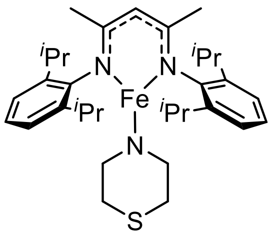

Fe-1 (80 mg, 0.14 mmol) was added to a J-Young NMR tube alongside C_6D_6 (0.6 mL). Thiomorpholine (15.86 µL, 0.14 mmol) was then added and an instant colour change was observed from yellow to red. The reaction was then heated to 80 °C for 18 h. Volatiles were then removed *in vacuo* and the resulting red residue was redissolved in a minimum of toluene and a few drops of pentane were added to the solution. Crystallisation of the solution at -30 °C for three days yielded yellow crystals of **Fe-4**. Drying the crystals *in vacuo* gave **Fe-4** as a yellow/brown powder (31 mg, 37%).

^{1}H NMR (500 MHz, 298 K, C_6D_6): 109.93 (γ-C*H*), -1.15 (thiomorpholine -CH_2), -6.50 (^{i}Pr-C*H*), -11.54 (backbone-CH_3), -15.82(^{i}Pr-C*H*), -20.00 (^{i}Pr-CH_3), -81.36 (*para*-Ar-*H*), -101.95 (*meta*-Ar-*H*), -119.73 (^{i}Pr-CH_3).

^{1}H NMR (500 MHz, 298 K, C_6D_6):

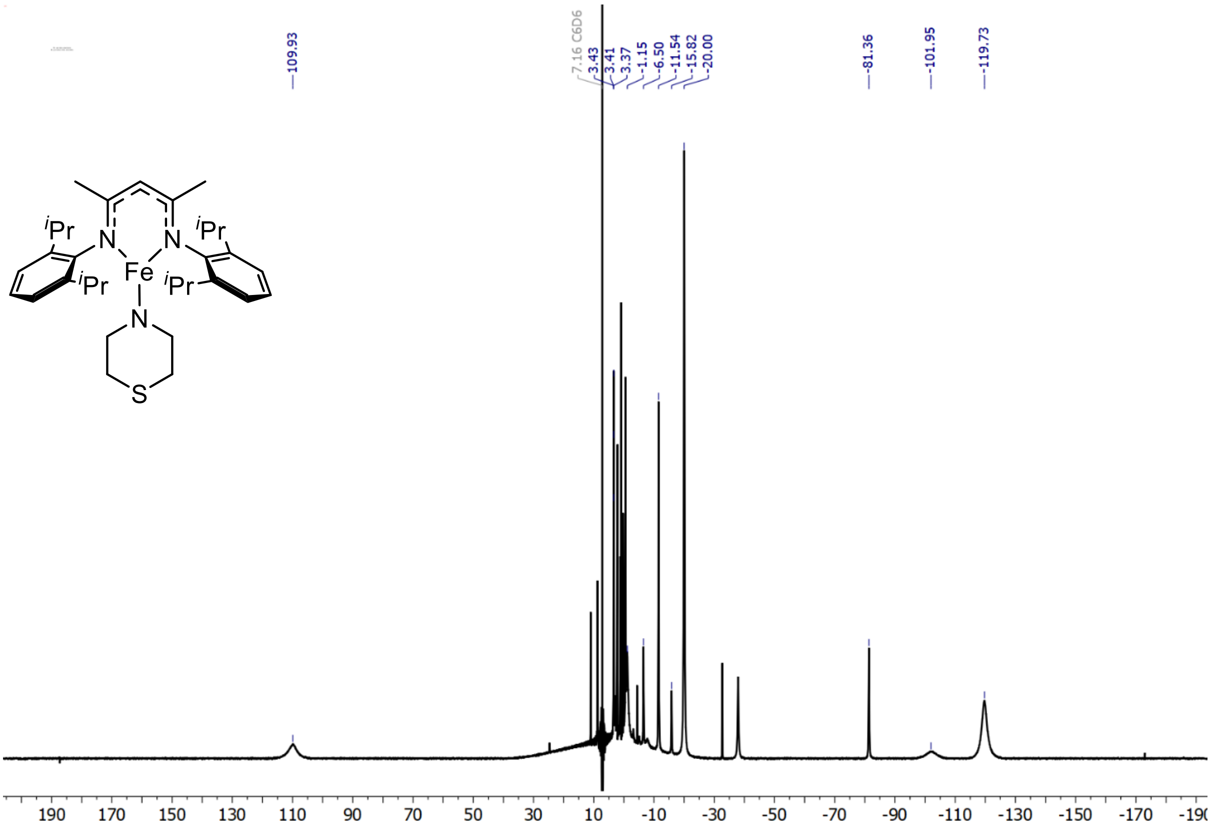

Figure S2: ^{1}H NMR (500 MHz, 298 K, C_6D_6) of **Fe-4.**

Fe-5:

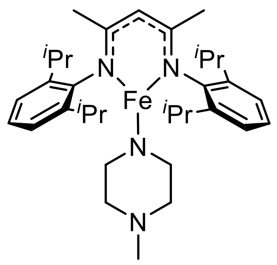

Fe-1 (80 mg, 0.14 mmol) was added to a J-Young NMR tube alongside C_6D_6 (0.6 mL). *N*-methylpiperazine (14.30 µL, 0.14 mmol) was then added and an instant colour change was observed from yellow to red. The reaction was then heated to 80 °C for 18 h. Volatiles were then removed *in vacuo* and the resulting red residue was redissolved in a minimum of pentane. Crystallisation of the solution at -30 °C for three days yielded deep red crystals of **Fe-5**. Drying the crystals *in vacuo* gave **Fe-5** as a red/brown powder (48 mg, 56%).

^{1}H NMR (500 MHz, 298 K, C_6D_6): 107.98 (γ-C*H*), 51.05 (piperazine-N-CH_3) -0.54 (^{i}Pr-C*H*), -6.59 (piperazine-CH_2), -11.34 (backbone-CH_3), -15.79 (^{i}Pr-C*H*), -19.79 (^{i}Pr-CH_3), -81.08 (*para*-Ar-*H*), -103.24 (*meta*-Ar-*H*), -118.12 (^{i}Pr-CH_3).

^{1}H NMR (500 MHz, 298 K, C_6D_6):

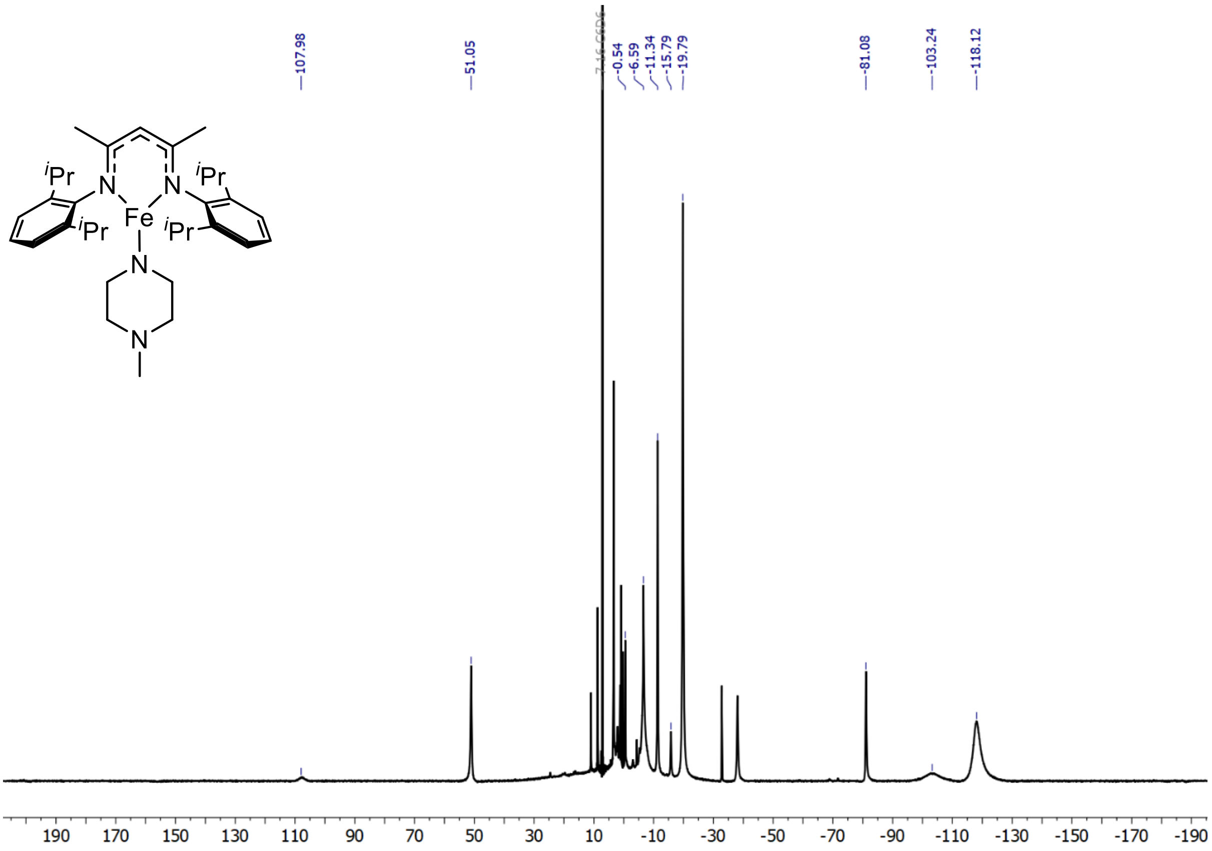

Figure S3: ^{1}H NMR (500 MHz, 298 K, C_6D_6) of **Fe-5.**

When excess *N*-methylpiperazine is added, **Fe-5'** forms:

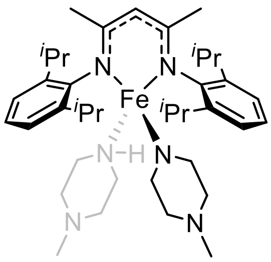

Fe-1 (80 mg, 0.14 mmol) was added to a J-Young NMR tube alongside C_6D_6 (0.6 mL). *N*-methylpiperazine (39.65 µL, 0.35 mmol) was then added and an instant colour change was observed from yellow to red. The reaction was then heated to 80 °C for 18 h. Volatiles were then removed *in vacuo* and the resulting red residue was redissolved in a minimum of pentane. Crystallisation of the solution at -30 °C for three days yielded deep red crystals of **Fe-5'**. Drying the crystals *in vacuo* gave **Fe-5'** as a red/brown powder (52 mg, 54%).

^{1}H NMR (500 MHz, 298 K, C_6D_6): 157.84 (coordinated piperazine CH_2), 105.58 (γ-C*H*), 83.20 (piperazine-N-CH_3), 50.46 (piperazine-N-CH_3) -1.33 (^{i}Pr-C*H*), -7.31 (piperazine-CH_2), -10.95 (backbone-CH_3), -15.79 (^{i}Pr-C*H*), -19.45 (^{i}Pr-CH_3), -80.31 (*para*-Ar-*H*), -101.97 (*meta*-Ar-*H*), -116.38 (^{i}Pr-CH_3).

^{1}H NMR (500 MHz, 298 K, C_6D_6):

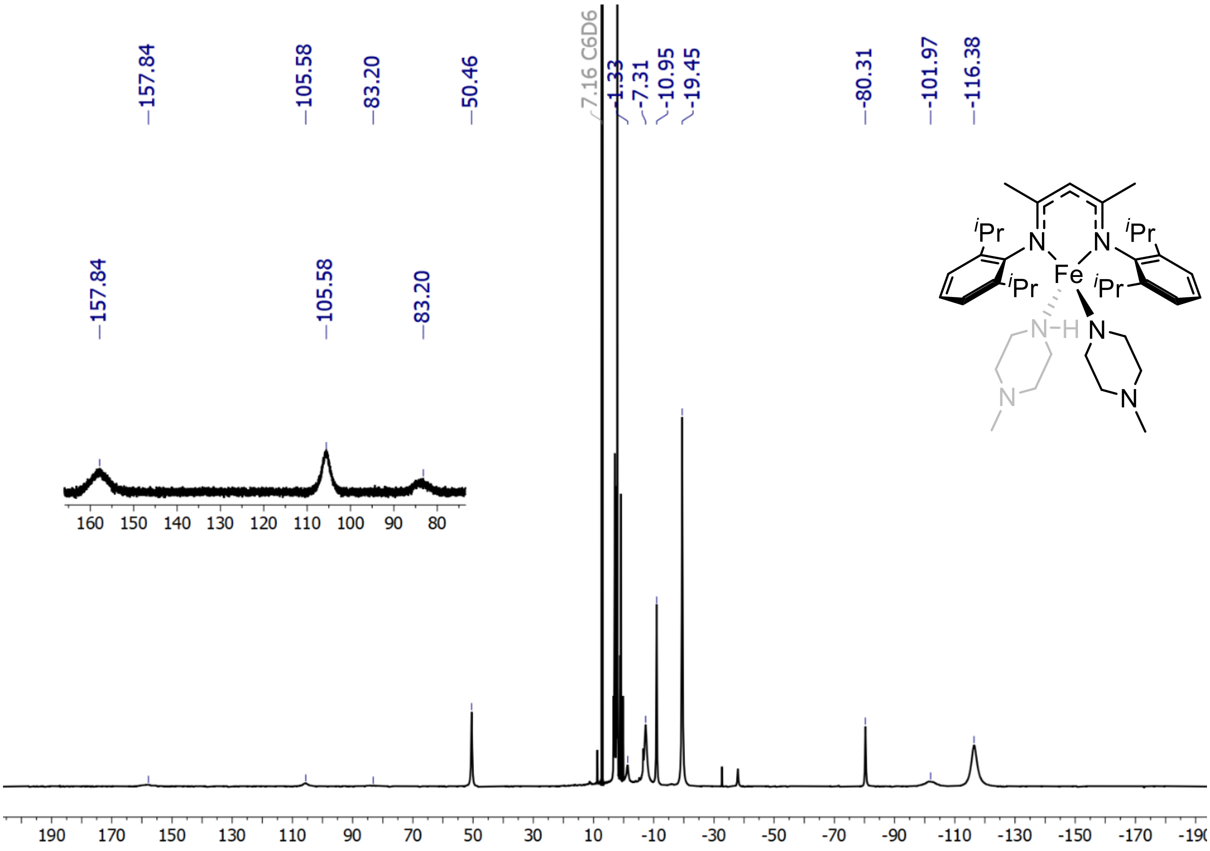

Figure S4: ^{1}H NMR (500 MHz, 298 K, C_6D_6) of **Fe-5'.**

Fe-6:

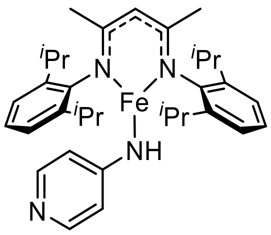

Fe-1 (80 mg, 0.14 mmol) was added to a J-Young NMR tube alongside 4-aminopyridine (13.1 mg, 0.14 mmol). Upon dissolution in toluene (0.6 mL), a red solution was formed. The reaction was then heated to 80 °C for one hour. The solution was then left to crystallise at -30 °C for three days, to yield deep red crystals of **Fe-6**. Drying the crystals *in vacuo* gave **Fe-6** as a red/brown powder (48 mg, 61%).

^{1}**H NMR** (500 MHz, 298 K, C_6D_6): 145.31, 36.27, 30.18, 17.92, 16.16, 15.53, 13.99, 4.30, -6.32, -10.04, -13.44, -15.12, -17.60, -21.89, -35.52, -37.93, -39.28, -68.75, -71.82, -74.41, -81.48, -84.31, -87.32.

^{1}**H NMR** (500 MHz, 298 K, C_6D_6):

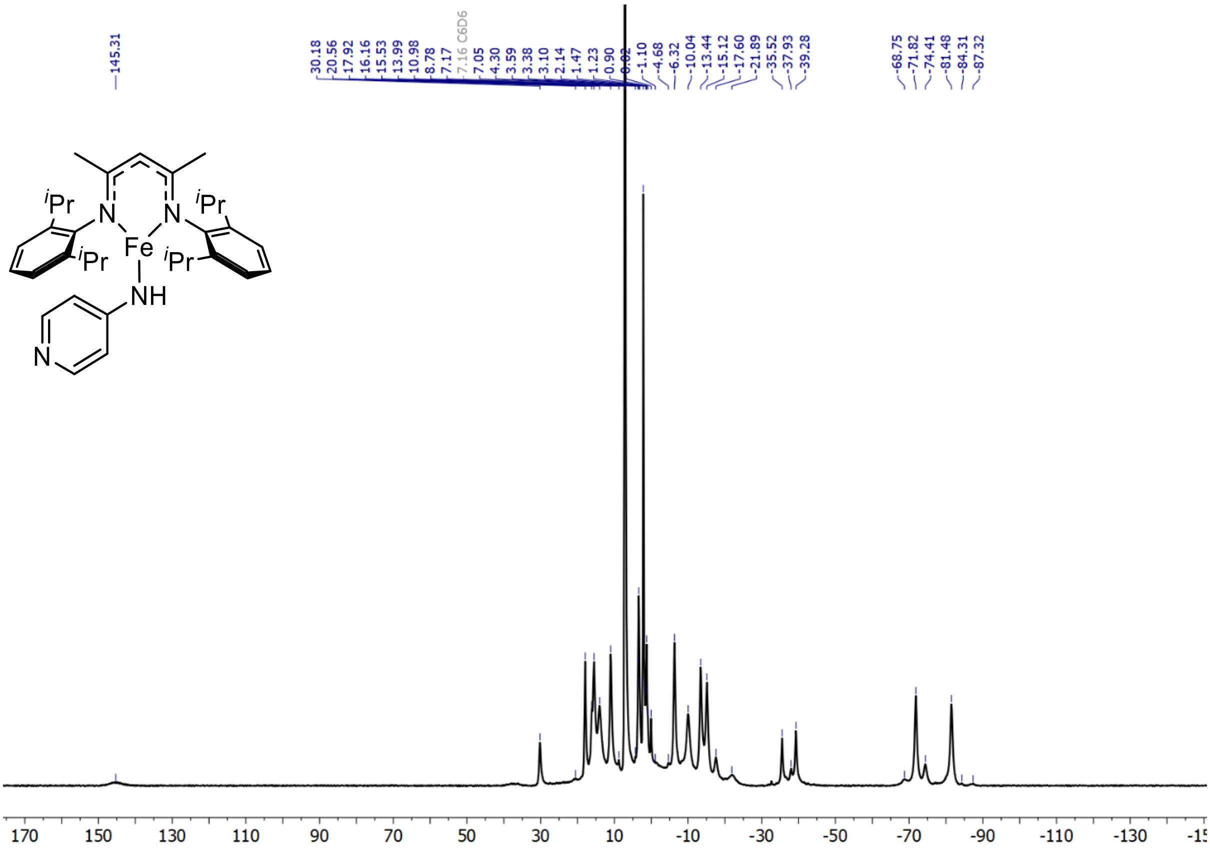

Figure S5: ^{1}**H NMR** (500 MHz, 298 K, C_6D_6) of **Fe-6.**

Fe-7:

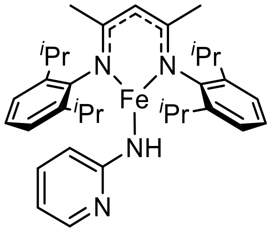

Fe-1 (80 mg, 0.14 mmol) was added to a J-Young NMR tube alongside 2-aminopyridine (13.1 mg, 0.14 mmol). Upon dissolution in toluene (0.6 mL), a red solution was formed. The reaction was then heated to 80 °C for one hour. The solution was then left to crystallise at -30 °C for three days, to yield deep red crystals of **Fe-7**. Drying the crystals *in vacuo* gave **Fe-7** as a red/brown powder (56 mg, 71%).

^{1}H NMR (500 MHz, 298 K, C_6D_6): 144.21, 67.60, 58.50, 44.00, 37.26, 35.61, 29.25, 19.05, 16.79, 16.60, 12.90, 12.47, 11.75, 11.70, 9.86, -2.28, -10.38, -12.83, -15.48, -17.33, -18.75, -20.99, -24.67, -25.52, -26.53, -28.11, -30.16, -31.69, -34.19, -39.40, -42.98, -43.81, -48.40, -50.74, -60.45.

^{1}H NMR (500 MHz, 298 K, C_6D_6):

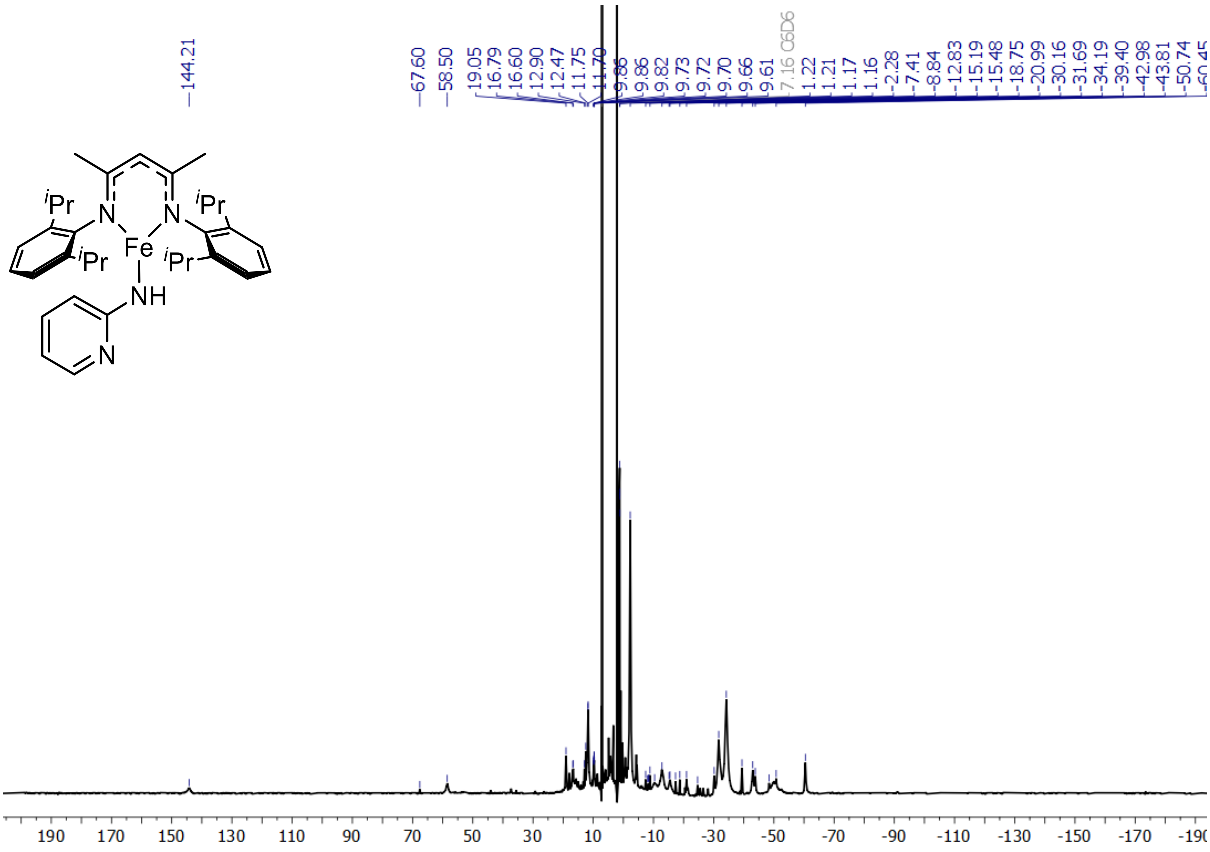

Figure S6: ^{1}H NMR (500 MHz, 298 K, C_6D_6) of **Fe-7.**

Fe-8:

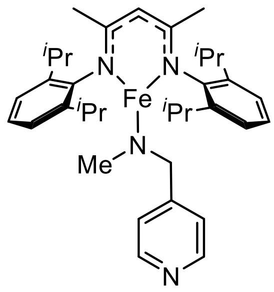

Fe-1 (80 mg, 0.14 mmol) was added to a J-Young NMR tube alongside C_6D_6 (0.6 mL). N-methylpyridine-4-methylamine (17.1 µL, 0.14 mmol) was then added and an instant colour change was observed from yellow to red. The reaction was then heated to 80 °C for 18 h. Volatiles were then removed *in vacuo* and the resulting red residue was redissolved in a minimum of toluene and a few drops of pentane were added to the solution. Crystallisation of the solution at -30 °C for three days yielded deep red crystals of **Fe-8**. Drying the crystals *in vacuo* gave **Fe-8** as a red/brown powder (32 mg, 38%).

^{1}H NMR (500 MHz, 298 K, C_6D_6): 21.56, 18.63, 8.74, 3.39, -0.53, -0.89, -1.74, -1.98, -5.15, -6.53, -7.81, -12.08, -32.64, -35.72, -37.89, -40.72, -44.62, -75.53, -87.63, -93.30, -95.29, -97.92.

^{1}H NMR (500 MHz, 298 K, C_6D_6):

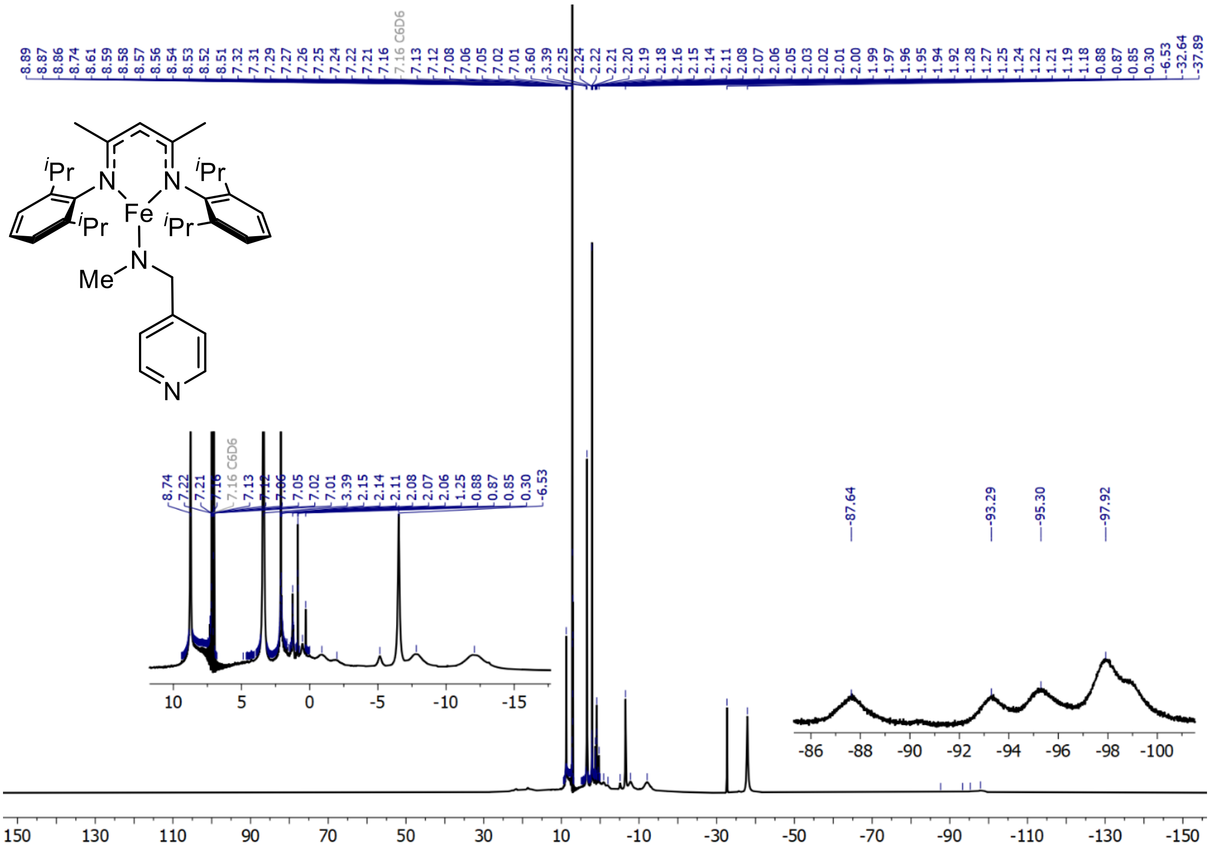

Figure S7: ^{1}H NMR (500 MHz, 298 K, C_6D_6) of **Fe-8.**

Fe-9:

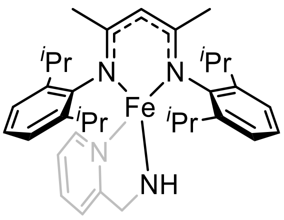

Fe-1 (80 mg, 0.14 mmol) was added to a J-Young NMR tube alongside C_6D_6 (0.6 mL). N-methylpyridine-4-methylamine (17.1 µL, 0.14 mmol) was then added and an instant colour change was observed from yellow to red. The reaction was then heated to 80 °C for 18 h. Volatiles were then removed *in vacuo* and the resulting red residue was redissolved in a minimum of pentane. Crystallisation of the solution at -30 °C for three days yielded deep red crystals of **Fe-9**. Drying the crystals *in vacuo* gave **Fe-9** as a red/brown powder (48 mg, 59%).

^{1}H NMR (500 MHz, 298 K, C_6D_6): 175.50, 80.06, 57.06, 42.54, 14.86, 4.22, -0.65, -6.49, -12.51, -32.67, -35.75, -37.92, -58.94, -66.24, -89.92.

^{1}H NMR (500 MHz, 298 K, C_6D_6):

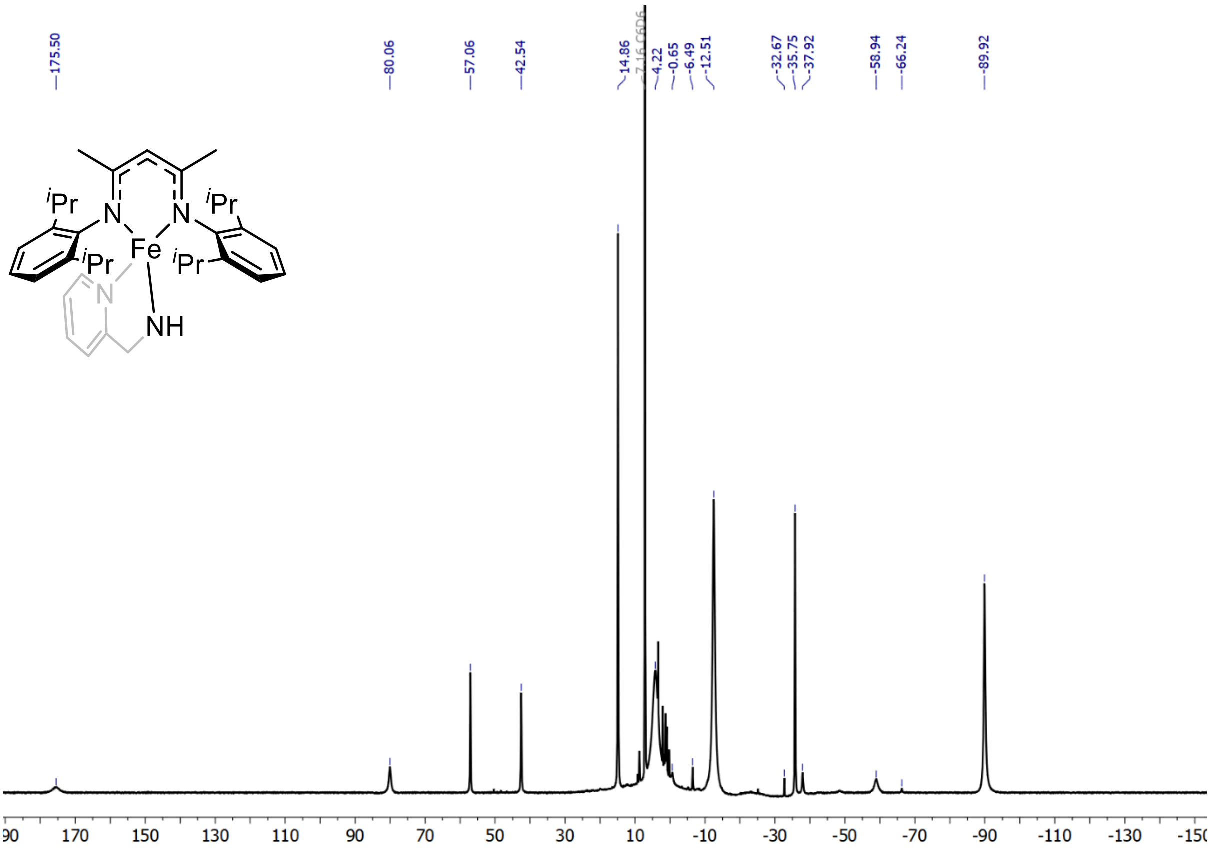

Figure S8: ^{1}H NMR (500 MHz, 298 K, C_6D_6) of **Fe-9.**

4. Table S1: Crystallographic Information

Identification code (CCDC)	**1962467**	**2211228**	**2211229**	**2211230**	**2211231**
Manuscript ID	**Fe-3**	**Fe-10**	**Fe-5**	**Fe-11**	**Fe-5'**
Empirical formula	$C_{38}H_{61}FeN_3O$	$C_{28.5}H_{37}FeN_3O$	$C_{34}H_{52}FeN_4$	$C_{26}H_{36}FeN_4$	$C_{156}H_{256}Fe_4N_{24}$
Formula weight	631.74	493.46	572.64	460.44	2691.23
Crystal system	cubic	orthorhombic	monoclinic	monoclinic	monoclinic
Space group	*Ia*-3*d*	*Pnma*	$P2_1/n$	$P2_1/c$	$P2_1/c$
a/ Å	45.3815(3)	15.3075(2)	12.89531(12)	17.9030(1)	10.4363(2)
b/ Å	45.3815(3)	17.3435(2)	20.10568(17)	18.0074(1)	22.7549(3)
c/ Å	45.3815(3)	19.3371(2)	13.23343(13)	14.8187(1)	16.6688(3)
α/ °	90	90	90	90	90
β/ °	90	90	98.3964(9)	92.814(1)	100.657(2)
γ/ °	90	90	90	90	90
U/ Å^3	93462.3(18)	5133.72(10)	3394.24(5)	4771.59(5)	3890.18(12)
Z	96	8	4	8	1
ρ_{calc}/ g cm^{-3}	1.078	1.277	1.121	1.282	1.149
μ/ mm^{-1}	3.316	4.895	3.745	5.206	3.351
F(000)	33024.0	2104.0	1240.0	1968.0	1464.0
Crystal size/ mm^3	0.18 × 0.072 × 0.062	0.320 × 0.150 × 0.100	0.208 × 0.181 × 0.12	0.156 × 0.121 × 0.046	0.152 × 0.131 × 0.058
2□ range for data collection/°	5.508 to 108.416	7.366 to 145.856	8.058 to 146.504	6.966 to 147.228	7.77 to 145.982
Index ranges	$-47 \le h \le 40$, $-40 \le k \le 43$, $-47 \le l \le 37$	$-17 \le h \le 18$, $-21 \le k \le 17$, $-22 \le l \le 23$	$-16 \le h \le 16$, $-23 \le k \le 24$, $-16 \le l \le 15$	$-22 \le h \le 22$, $-22 \le k \le 22$, $-18 \le l \le 17$	$-10 \le h \le 12$, $-28 \le k \le 28$, $-20 \le l \le 20$
Reflections collected	120773	47865	46504	89893	69003
Independent reflections, R_{int}	4756, 0.1490	5299 ,0.0606	6808 ,0.0272	9584 ,0.0393	7746 ,0.0592
Data/restraints/parameters	4756/0/353	5299/0/332	6808/0/363	9584/0/573	7746/0/427
Goodness–of–fit on F^2	1.057	1.042	1.071	1.084	1.020
Final $R1$, $wR2$ [I>=2σ(I)]	0.0588, 0.1234	0.0416, 0.1070	0.0676, 0.1612	0.0336, 0.0823	0.0413, 0.0972
Final $R1$, $wR2$ [all data]	0.1206, 0.1429	0.0496, 0.1121	0.0699, 0.1664	0.0373, 0.0840	0.0525, 0.1026
Largest diff. peak/hole/ e Å^{-3}	0.17/−0.25	0.77/−0.54	0.90/−0.24	0.32/−0.43	0.64/−0.25

Identification code (CCDC)	**2211232**	**2211233**	**2211234**	**2211235**	**2211236**
Manuscript ID	**Fe-4**	**Fe-6**	**Fe-7**	**Fe-8**	**Fe-9**
Empirical formula	$C_{33}H_{49}FeN_3S$	$C_{143}H_{190}Fe_4N_{16}$	$C_{37.5}H_{50}FeN_4$	$C_{148}H_{202}Fe_3N_{12}$	$C_{35}H_{48}FeN_4$
Formula weight	575.66	2356.50	612.66	2316.75	580.62
Crystal system	monoclinic	monoclinic	monoclinic	triclinic	orthorhombic
Space group	$P2_1/n$	$P2_1/n$	$P2_1/n$	*P*-1 (No. 2)	$Pna2_1$
a/ Å	8.3823(1)	22.1561(8)	13.1844(2)	17.5946(5)	16.4239(6)
b/ Å	18.8041(2)	25.1015(5)	17.1179(2)	20.3188(6)	12.4296(5)
c/ Å	20.7623(2)	28.6880(9)	15.3992(2)	21.5881(4)	15.7961(8)
α/ °	90	90	90	94.368(2)	90
β/ °	90.009(1)	90.268(3)	98.9580(10)	101.648(2)	90
γ/ °	90	90	90	113.852(3)	90
U/ $Å^3$	3272.59(6)	15954.7(8)	3433.04(8)	6805.1(3)	3224.7(2)
Z	4	4	4	2	4
ρ_{calc}/ g cm^{-3}	1.168	0.981	1.185	1.131	1.196
μ/ mm^{-1}	4.460	3.203	3.740	2.925	3.953
F(000)	1240.0	5056.0	1316.0	2504.0	1248.0
Crystal size/ mm^3	0.34 × 0.22 × 0.114	0.332 × 0.136 × 0.068	0.179 × 0.112 × 0.021	0.268 × 0.194 × 0.159	0.174 × 0.115 × 0.028
2□ range for data collection/°	8.518 to 146.47	7.098 to 136.502	7.776 to 146.266	6.706 to 146.572	8.922 to 148.004
Index ranges	$-8 \le h \le 10$, $-22 \le k \le 23$, $-25 \le l \le 25$	$-26 \le h \le 26$, $-17 \le k \le 30$, $-34 \le l \le 34$	$-16 \le h \le 16$, $-21 \le k \le 21$, $-17 \le l \le 19$	$-21 \le h \le 18$, $-18 \le k \le 25$, $-26 \le l \le 26$	$-20 \le h \le 18$, $-15 \le k \le 15$, $-19 \le l \le 19$
Reflections collected	43322	115193	62093	95954	45464
Independent reflections, R_{int}	6580 ,0.0406	28590 ,0.0996	6859 ,0.0518	27086 ,0.0539	6491 ,0.0926
Data/restraints/parameters	6580/0/354	28590/0/1445	6859/134/434	27086/0/1269	6491/2/379
Goodness–of–fit on F^2	1.038	0.981	1.021	1.046	1.024
Final $R1$, $wR2$ [I>=2σ(I)]	0.0275, 0.0719	0.0647, 0.1541	0.0381, 0.0936	0.0442, 0.1062	0.0421, 0.0974
Final $R1$, $wR2$ [all data]	0.0287, 0.0730	0.1107, 0.1758	0.0473, 0.0989	0.0594, 0.1128	0.0525, 0.1045
Largest diff. peak/hole/ e $Å^{-3}$	0.29/−0.25	0.49/−0.39	0.62/−0.42	0.35/−0.26	0.23/−0.30
Flack parameter	–	–	–	–	−0.014(3)

Data for **CCDC 1962467, 2211228, 2211229, 2211230, 2211231, 2211232, 2211233, 2211234, 2211235** and **2211236** were collected at 150 K using an Agilent SuperNova diffractometer, a Cu-K□ source. Refinement was achieved using SHELXL via the Olex2 interface.

Convergences were achieved smoothly with the exception of that for **2211232**. Noteworthy points of note pertaining to the crystallographic experiments will now be detailed.

The beautiful molecule in compound **1962467** crystallised in Space Group 230! The asymmetric unit therein equates to one-sixth of a hexamer and a region of solvent. In many ways, attaining this refinement is a tribute to modern day diffractometers because the solvent is very disordered. As a result, in this high symmetry space group, the diffraction intensities faded to almost zilch at higher Bragg angles. Ultimately, the data were truncated to a resolution of 0.95Å for refinement purposes, and even at this value, the R_{int} for the dataset bears the scars of diffraction intensity fall-off. The solvent could not be readily modelled and, hence, was treated *via* the solvent mask algorithm available in Olex-2, with an allowance for one molecule of pentane per asymmetric unit made in the formula as presented. The hexamer can be generated from the monomer by virtue of the 3-fold rotary-inversion axis implicit in the space group.

The asymmetric unit in the structure of **2211228** comprises half of a dimer molecule and a toluene fragment with half site-occupancy. Atoms Fe1, Fe2, N1, N2, O1, O2, C3, and C15 plus the solvent are coincident with a crystallographic mirror plane with serves to generate the remainder of the dimer. The methyl hydrogens in the toluene are crystallographically disordered about this mirror plan as well.

Two crystallographically independent dimer halves constitute the asymmetric unit in the structure of **2211230**. Both fragments lie proximate to inversion centres that are intrinsic to the space group symmetry, which serve to complete the molecule in each instance.

A very satisfying refinement was ultimately achieved for **2211232,** which is presented herein. However, this was only attained by disentangling 40% twinning by pseudo-merohedry (2-fold rotation about *b*). This type of twinning is often observed for crystals where the cell dimensions mimic a crystal system with higher-symmetry than that which is truly present. In this case, the β angle close to 90° plus the twinning superficially rendered the data representing orthorhombic symmetry. Indeed, with appropriate axes swaps, poor but recognisable solutions could be brokered in *Pnma* (systematic weakness of data pertaining to the *a*-glide, which was not really present) and *Pnm*21. However, it was evident that there was much amiss in both cases with *R*1 values remaining high and multiple ADPs that gave rise to an appalling vista. Once twinning was addressed and the data accordingly integrated to reflect the monoclinic symmetry, the emergent solution and refinement was exquisite, crystallographically.

The asymmetric unit in the structure of **2211233** equates to one tetramer and some disordered solvent. The latter, as the model was refined, provided some insight into why a large sample was needed for this data collection (in order to broker some decent data) and why diffraction intensity declined rather steeply at high Bragg angles. These observations reflect that there are approximately four molecules of toluene per asymmetric unit in the structure, bedeviled by disorder in varying degrees. As such, solvent

was ultimately treated using the solvent mask algorithm available in Olex-2, and an allowance was made for same in the formula as presented. Data were truncated at a resolution of 0.83 Å because of the declining diffraction intensity, a legacy of which is evident in the R_{int} value. Despite these shortcomings, the chemical characterisation of the species is unequivocal.

In **2211234**, the asymmetric unit comprises half of one dimer molecule and a region of solvent. The hydrogen attached to N3, in the former, was located and refined at a distance of 0.98Å from the parent atom. The solvent moiety suffered from disorder, some localized to the molecule and some by virtue of straddling a crystallographic inversion-centre that is implicit to the space group. Overall, there is half of one molecule of toluene present per asymmetric unit which was modelled (with the assistance of the excellent FragmentDB plugin for Olex2, which is a GUI-specific implementation of the excellent DSR refinement package[6]) as a full molecule with half site-occupancy, wherein the methyl group was disordered over two positions in equal measure.

The asymmetric unit in the structure of **2211235** equates to one molecule of the iron complex, two ordered molecules of toluene, three disordered molecules of toluene and one disordered molecule of pentane. Disordered solvent was addressed *via* the solvent mask algorithm available in Olex-2, but allowance was made for same in the formula as presented.

Finally, the nitrogen bound hydrogen was located and refined at a distance of 0.9 Å from the parent atom in the structure of **2211236**.

Crystallographic data for all compounds have been deposited with the Cambridge Crystallographic Data Centre as supplementary publications CCDC **1962467** and CCDC **2211228-2211236**. Copies of these data can be obtained free of charge on application to CCDC, 12 Union Road, Cambridge CB2 1EZ, UK [fax(+44) 1223 336033, e-mail: deposit@ccdc.cam.ac.uk.

5. References

[1] a) Sciarone, T. J. J.; Meetsma, A.; Hessen, B. *Inorg. Chim. Acta*, **2006**, *359*, 1815-1825; b) Linford-Wood, T. G.; Mahon, M. F.; Grayson, M. N.; Webster, R. L. *ACS. Catal.* **2022**, *12*, 2979-2985.

[2] Brunauer, S.; Emmett, P. H.; Teller, E. *J. Am. Chem. Soc.* **1938**, *60*, 309-319.

[3] Barrett, E. P.; Joyner, L. G.; Halenda, P. P. *J. Am. Chem. Soc.* **1951**, *73*, 373-380.

[4] Evans, R.; Dal Poggetto, G.; Nilsson, M.; Morris, G. A. *Anal. Chem.* **2018**, *90*, 3987-3994.

[5] Evans, R.; Deng, Z.; Rogerson, A. K.; McLachlan, A. S.; Richards, J. J.; Nilsson, M.; Morris,G. A. *Angew. Chem. Int. Ed.* **2013**, *52*, 3199-3202.

[6] Kratzert, D.; Holstein, J.J; Krossing, I. *J. Appl. Cryst*. **2015**, *48*, 933-938.
